# A Novel Prognostic Tool for Glioma Based on Enhancer RNA-Regulated Immune Genes

**DOI:** 10.3389/fcell.2021.798445

**Published:** 2022-01-20

**Authors:** Wei Tian, Kegong Chen, Guangcan Yan, Xinhao Han, Yanlong Liu, Qiuju Zhang, Meina Liu

**Affiliations:** ^1^ Department of Biostatistics, School of Public Health, Harbin Medical University, Harbin, China; ^2^ Department of Cardio-Thoracic Surgery, The Second Affiliated Hospital of Harbin Medical University, Harbin, China; ^3^ Department of Colorectal Surgery, Harbin Medical University Cancer Hospital, Harbin, China

**Keywords:** enhancer RNA, super enhancer RNA, immune-related gene, prognosis, glomas

## Abstract

**Background:** Gliomas are the most malignant tumors of the nervous system. Even though their survival outcome is closely affected by immune-related genes (IRGs) in the tumor microenvironment (TME), the corresponding regulatory mechanism remains poorly characterized.

**Methods:** Specific enhancer RNAs (eRNAs) can be found in tumors, where they control downstream genes. The present study aimed to identify eRNA-regulated IRGs, evaluate their influence on the TME, and use them to construct a novel prognostic model for gliomas.

**Results:** Thirteen target genes (ADCYAP1R1, BMP2, BMPR1A, CD4, DDX17, ELN, FGF13, MAPT, PDIA2, PSMB8, PTPN6, SEMA6C, and SSTR5) were identified and integrated into a comprehensive risk signature, which distinguished two risk subclasses. Discrepancies between these subclasses were compared to explore potential mechanisms attributed to eRNA-regulated genes, including immune cell infiltration, clinicopathological features, survival outcomes, and chemotherapeutic drug sensitivity. Furthermore, the risk signature was used to construct a prognostic tool that was evaluated by calibration curve, clinical utility, Harrell’s concordance index (0.87; 95% CI: 0.84–0.90), and time-dependent receiver operator characteristic curves (AUCs: 0.93 and 0.89 at 3 and 5 years, respectively). The strong reliability and robustness of the established prognostic tool were validated in another independent cohort. Finally, potential subtypes were explored in patients with grade III tumors.

**Conclusion:** Overall, eRNAs were associated with immune-related dysfunctions in the TME. Targeting of IRGs regulated by eRNAs could improve immunotherapeutic/therapeutic outcomes.

## Background

Gliomas are the most common malignant tumors of the central nervous system, accounting for more than 80% of cases, and have complex survival outcomes (Lin et al., 2017; Bouckaert et al., 2020). Existing therapeutic strategies and regimens have proven inadequate (Qin et al., 2017). In recent years, immunotherapy has emerged as a promising approach for treating patients with malignant tumors including gliomas (Jahan et al., 2018). However, the clinical benefits may outweigh the costs only in a small fraction of glioma patients, due to the “cold tumor” character and complex tumor microenvironment (TME) of this malignancy (Wang et al., 2020; Cai et al., 2021). Although several studies have revealed a function for immune-related genes (IRGs) in immune cell infiltration, tumorigenesis, and tumor prognosis (Najima et al., 2016), their exact role and regulators remain unclear (Lou et al., 2020). Diverse and complex factors influence IRGs, including somatic mutations, variations in gene copy number, and gene methylation (Binder et al., 2019; Huang et al., 2019). Because these effects are not always consistent and identical between IRGs, the regulatory relationship has been difficult to ascertain and some important immune modulators may have been overlooked. Therefore, more specific immune modulators should be identified, and the functions of their target genes should be investigated to better understand the underlying mechanisms.

Enhancers are important distal regulatory DNA elements. They are transcribed bidirectionally to produce enhancer RNAs (eRNAs) and directly drive tumorigenesis (Lee et al., 2020). Besides possessing enhancer activity, eRNAs can also regulate clinically relevant genes and immune checkpoints. Accordingly, eRNAs may play an important role in controlling immune-related functions and could promote clinical antitumor therapies by interacting with transcription factors (Hsieh et al., 2014; Schaukowitch et al., 2014; Liang et al., 2016; Ye et al., 2018; Zhang et al., 2019). In addition to conventional eRNAs, there are special eRNAs transcribed from more than one enhancer in a series and referred to as super-enhancer RNAs (seRNAs) (Pott and Lieb, 2015; Peng et al., 2019). SeRNAs tend to have stronger transcriptional activity than conventional eRNAs, suggesting a greater role in the regulation of gene expression (Thandapani, 2019). Crucially, because eRNA expression is cell type-specific, they and their target genes could serve as diagnostic tumor biomarkers and therapeutic targets. At present, though, it remains to be determined how eRNAs/seRNAs affect immune function and influence survival outcomes in glioma patients.

In this study, we aimed to identify survival-mediating IRGs regulated by eRNAs/seRNAs and explore potential mechanisms causing immune malfunction in the TME of gliomas. The identified IRGs were used to construct a prognostic tool to predict survival outcomes, as well as facilitate clinical decisions and individual management. The obtained information could be used to improve the therapeutic regimen and prolong the overall survival (OS) of glioma patients.

## Methods

### Data Access and Patient Selection

Gene expression, copy number variance, methylation (450K) data and phenotype data for the training cohort were downloaded from The Cancer Genome Atlas (TCGA) dataset using the Xena Cancer Browser (https://xena.ucsc.edu/). The external validation cohort was obtained from the Chinese Glioma Genome Atlas (CGGA; https://www.cgga.org.cn/). Enhancer expression data and the regulatory information for eRNAs and their target genes in TCGA patients were acquired from the Enhancer RNA in Cancers database (https://hanlab.uth.edu/eRic/) (Zhang et al., 2019). The immune gene list was available from ImmPort (https://www.immport.org).

Patients were recruited according to the following criteria: 1) glioma as primary tumor and 2) OS > 30 days. Patients with missing information were excluded and those with an incomplete pattern were subjected to missing completely at random testing using Little’s method (Adamis et al., 2020). These criteria and statistical analyses were applied to both TCGA and CGGA data. The batch effect was corrected using the ComBat method (Johnson et al., 2007).

### Identification of eRNA-Regulated Immune-Related Genes and Functional Enrichment Analysis

Prognostic eRNAs were obtained after Kaplan-Meier and univariate Cox analyses, with adjusted *p* < 0.05 as the cut-off criterion. The selected prognostic eRNAs were matched to their target genes including IRGs. Kaplan-Meier and Spearman correlation analyses between the target gene and eRNA expression were carried out simultaneously to identify IRGs related to OS and eRNAs. The correlation filtration process was based on whether the Spearman correlation coefficient (*r*_*s*_) was >0.3. The biological function of IRGs was revealed by Gene Ontology (GO) and Kyoto Encyclopedia of Genes and Genomes (KEGG) enrichment analyses.

### Construction and Evaluation of the Immune-Related Gene Signature

The least absolute shrinkage and selection operator (LASSO) Cox regression model was used to narrow the list of IRGs to the most important ones. The risk score was calculated using the inner products between the LASSO Cox regression coefficients and their gene expression levels. The time-dependent receiver operator characteristic (ROC) curve and respective area under the curve (AUC) were applied to determine the specificity and sensitivity of the IRG signature in predicting survival outcomes. The turning point of the risk score in the density curve was selected as the cut-off for separating patients into low- and high-risk groups in the TCGA cohort. The difference in survival between low- and high-risk patients was evaluated using the Kaplan-Meier curve. The IRG landscape and statistical comparisons of clinical phenotypes were used to reveal the relationship between IRGs and clinicopathological characteristics. The same methods were applied to an independent validation cohort.

### Comparison of Immune Landscape, Pathway Activity and Therapeutic Sensitivity

To investigate the clinical heterogeneity between the two risk groups, differences in immune landscape, pathway activity, and therapeutic sensitivity were compared.

The proportion of immune and tumor cells in each glioma patient was evaluated with the estimate package in R. To further characterize the TME and predict the immune cell infiltration status, a computational algorithm was run using the CIBERSORT package in R. After excluding samples with *p* > 0.05, the infiltration proportion of 22 immune cells was compared between the low- and high-risk groups for each glioma patient. Single-sample gene set enrichment analysis (ssGSEA) was implemented to obtain a second estimate of immune cell infiltration (Barbie et al., 2009). Other immunocyte infiltration algorithms were also taken into account in our research, including xCell and Tumor Immune Estimation Resource (TIMER) (Aran et al., 2017; Li et al., 2017; Cai et al., 2021). As an important gene family related to immune functionality, human leukocyte antigen (HLA) was compared to uncover additional differences in the TME.

Gene set variation analysis (GSVA) was applied to explore differences in pathway activity between the low- and high-risk groups. KEGG pathway activity was estimated, compared, and visualized between low- and high-risk groups based on the GSVA and limma packages in R.

Immune checkpoint expression levels and tumor mutation burden (TMB) were used to predict immunotherapeutic efficacy for patients with different immune risk signatures. Checkpoint expression of cytotoxic T-lymphocyte-associated protein 4 (CTLA-4) and programmed cell death ligand-1 (PD-L1) served as the main biomarker for anti-CTLA-4 and anti-PD-L1 immunotherapy. To identify potential drugs against gliomas, the half-maximal inhibitory concentration (IC_50_) for various chemotherapeutic agents, including cisplatin, bleomycin, docetaxel, doxorubicin, gemcitabine, and paclitaxel, was predicted. The estimation was predicted by the *pRRophetic* package which was based on the ridge regression analysis and the Cancer Genome Project (CGP) database, and directly used the expression matrix of all genes without any data processing (Geeleher et al., 2014). The IC50 of temozolomide could be predicted by the *oncoPredict* package, based on the Genomics of Drug Sensitivity in Cancer database (Maeser et al., 2021).

### Development and Validation of the Prognostic Model

Considering the risk signature and traditional clinical variables, univariate and multivariate Cox models were employed to select reliable prognostic features, including age, sex, radiotherapy status, isocitrate dehydrogenase (IDH) status, and X1p19q co-deletion status, with which to construct a prognostic model. The indicator age was transformed into a dummy variable (young and elderly) according to its median. In multivariate Cox analysis, a stepwise process was used to confirm crucial characteristics. Harrel’s concordance index and AUCs were calculated, and the calibration, Kaplan-Meier, ROC, and decision analysis curves were plotted to assess sensitivity, specificity, and clinical utility in predicting survival outcomes using the prognostic model. These indicators were also evaluated in an independent CGGA cohort.

### Exploring the Relationship Between Risk Signature and Genomic Alterations

According to the Consortium to Inform Molecular and Practical Approaches to CNS Tumor Taxonomy, genomic events, such as telomerase reverse transcriptase promoter (TERTp) mutation, the combination of whole chromosome 7 gain and whole chromosome 10 loss (chr 7+/10-), epidermal growth factor receptor amplification, and O-6-methylguanine-DNA-methyltransferase promoter (MGMTp) methylation status, are closely related to survival outcomes (Geisenberger et al., 2015; Brat et al., 2018). These markers could not be used to establish the prognostic model in this study, as that would jeopardize validation in the CGGA cohort. However, the relationship between them and the risk signature was analyzed to explore a more comprehensive potential network. In line with earlier reports (Galbraith et al., 2020), the detection rate of TERTp mutations was too low to allow direct evaluation of its prognostic value in the TCGA cohort. To overcome this problem, we used TERT gene expression as a proxy for TERTp mutation status (Galbraith et al., 2020). Traditionally, MGMTp methylation status is an important common biomarker, but the TCGA cohort could not directly provide relevant information, compelling the use of MGMT methylation level to represent MGMTp status (Cai et al., 2021).

### Identification of Subtypes in Different Tumor Grades

By applying the risk signature to tumor grade data, we sought to explore the relationship between the signature and tumor severity, which could identify valuable subtypes. The reliability of subtype information was assessed in the TCGA cohort and verified in the CGGA cohort. Differentially expressed genes were identified, and the potential pathways underscoring biological differences were probed by ssGSEA.

### Estimation of Influence in Gene Expression by eRNA

To evaluate the contribution of eRNA in the regulation of gene expression, we constructed a comprehensive linear model in which the covariate variables were taken into account, including age, grade, gender, radiotherapy, IDH mutation, 1p19q codeletion, gene methylation, CNV, and eRNA. The raw methylation data were transformed as beta values for each gene. The partially explained sum of squares (ESS) that could be used to explain how much of the variance was attributed to the independent factor, was the main indicator to evaluate eRNA.

### Cell Culture and Transfection

U251 and LN229 were the human glioma cell lines, commercially purchased from the Cell Bank of the Chinese Academy of Sciences (Beijing, China). U251 cell was cultured in DMEM-6429 (Sigma, MO, United States) containing 5% fetal bovine serum (FBS, HyClone, Logan, UT, United States), while LN229 cell was cultured in DMEM-5546 (Sigma, MO, United States) containing 10% fetal bovine serum (FBS, HyClone, Logan, UT, United States). siRNAs were obtained from the company of Nantong Biomics Biotechnologies. The siRNA sequences targeting PTPN6 and PSMB8 in this study included: si-PTPN6-1, si-PTPN6-2: and negative controls (si-PTPN6-NC); si-PSMB8-1, si-PSMB8-2 and negative controls (si-PSMB8-NC), respectively. The sequences all were listed in Supplementary Table S1. Lipofectamine 3,000 (Thermo Fisher Scientific, Inc.) was used for transfection according to the manufacturer’s instructions. The cells were harvested 48 h after transfection.

### Protein Extraction and Western Blot Analysis

Total proteins were extracted from the cells with RIPA buffer and quantified by a BCA kit (Beyotime Biotechnology). About 30 μg of extracted proteins were separated by SDS-PAGE and then transferred to PVDF membranes (Merck Millipore). Soaked with 5% non-fat milk for 2 h at 25°C and incubated with PTPN6 (Abcam; 1:1,000; ab124942), PSMB8 (Abcam; 1:1,000; ab180606) and GAPDH (Abcam; 1:5,000; ab9485), the PVDF membranes were incubated with a secondary antibody (Cell Signaling Technology).

### Cell Viability and Colony Formation Assays

EdU staining was used to assess the cell proliferation through a commercial EdU Kit (UE, China) according to the manufacturer’s protocols. Images were obtained using a fluorescence microscope (Leika, Germany) and analyzed with ImageJ. The colony formation assay was carried out to detect the clonogenic capacity. The transfected U251 and LN229 cells were seeded into 35 mm culture dishes at a concentration of 1,000 cells per dish and cultivated for 8 and 14 days, respectively. Cell colonies were fixed with paraformaldehyde and stained with 0.1% crystal violet (Beyotime) for 20 min, whose colony counting was determined by microscope.

### Transwell Assay

Cell invasion was evaluated by performing the Chamber matrigel invasion 24-well units (Costar) according to the manufacturer’s instructions. The transfected cells were suspended in a serum-free medium and 1 × 105 cells per chamber were plated into the upper chamber of the transwell system with a pore size of 8 µm. The bottom chamber was filled with a medium containing 10% FBS. After incubation for 24 h, the migrated/invaded cells in the lower chamber (below the filter surface) were fixed in 4% paraformaldehyde, stained with crystal violet solution, and counted under a microscope.

### Evaluation of Cell Apoptosis

Cell apoptosis was determined by Annexin V-FITC/PI Apoptosis Detection Kit (BD Pharmingen, United States) by flow cytometry. 1 × 105 cells of each group were harvested and resuspended in 300 μl binding buffer containing 5 μl Annexin V-FITC for 30 min at 4°C in the dark, followed by further incubation with 5 μl PI for 5 min. Samples were analyzed with a FACSCanto II equipped with FACSDiva software (BD Bioscience). Live cells were identified as Annexin V-FITC-/PI- (lower left quadrant), apoptotic cells as Annexin V-FITC+/PI+ (upper right quadrant).

### Knockdown of eRNA Using ASO

Locked nucleic acid (LNA)-modified ASOs complementary to eRNA of PTPN6 and PSMB8 were designed and purchased from GenePharma (Shanghai, China). The sequences are listed in Supplementary Table S2. For the transfection of U251 and LN229 cells, ASOs were mixed with Lipofectamine 3,000 in serum-free DMEM (Sigma) according to the manufacturer’s protocols. At varying concentrations of ASOs, dissolved DMEM was added, and the cells were incubated in a growth medium for 4 h at 37°C and 5% CO_2_. At 48 h after transfection, the cells were harvested for further analysis.

### The Human Protein Atlas

The Human Pathology Atlas project (https://www.proteinatlas.org) contains immunohistochemistry (IHC) data using a tissue microarray-based analysis on the different normal tissue types, and proteome analysis of the major cancer types. Staining intensity, quantity, location, and patients’ information in patients with the respective types of cancer were available online. In this study, protein levels of PTPN6 and PSMB8 in glioma tissues and normal tissues analyzed with the IHC method were analyzed at Human Protein Atlas.

### Statistical Analysis

All statistical analyses were performed using R software (https://www.r-project.org; version 4.0.3) and were two-sided. Results with *p* < 0.05 were considered statistically significant. The main statistical method used in this study included the Wilcoxon test, correlation test, chi-square test, Little’s test, and proportional hazards assumption test.

## Results

### Selection of eRNA-Regulated Immune-Related Genes

**FIGURE 1:**
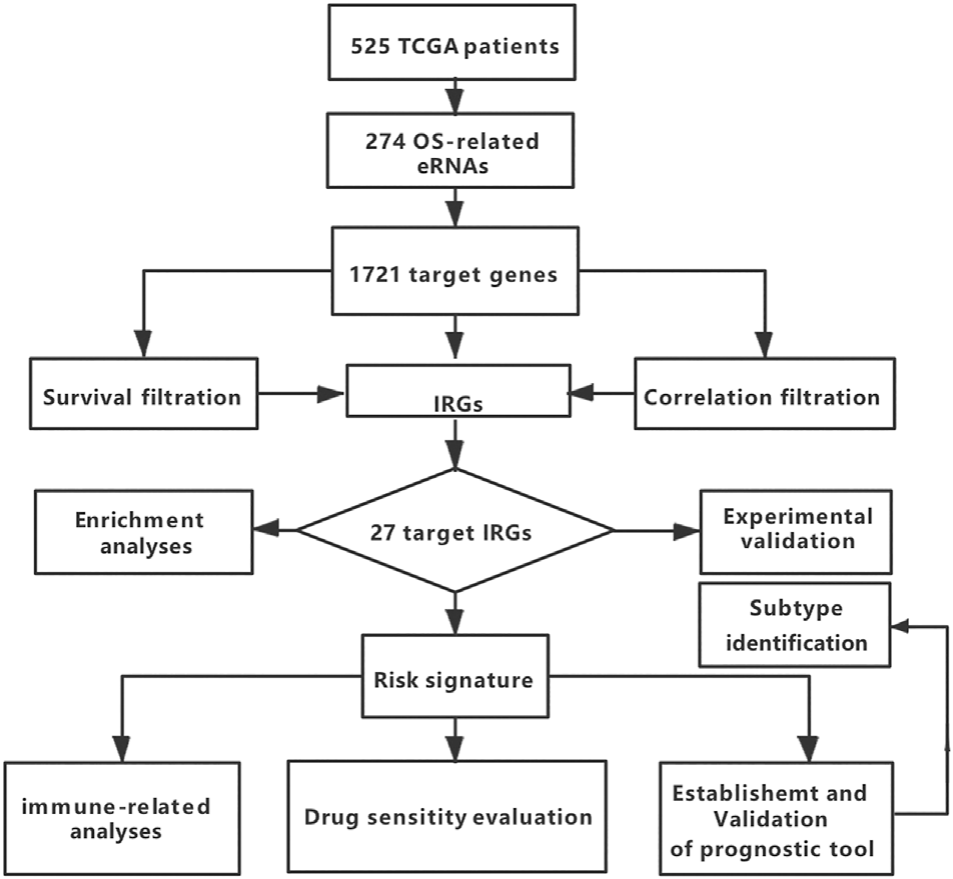
Flowchart of the study.

A total of 702 TCGA patients were selected as the training cohort, and 1018 CGGA patients with gliomas were used as the validation cohort. After adjusting for incomplete information, 525 TCGA (*p* = 0.11) and 513 CGGA (*p* = 0.13) glioma patients were included. Their clinicopathological characteristics are reported in Supplementary Tables S3, S4. A flowchart of this study is summarized in Figure 1.

**FIGURE 2:**
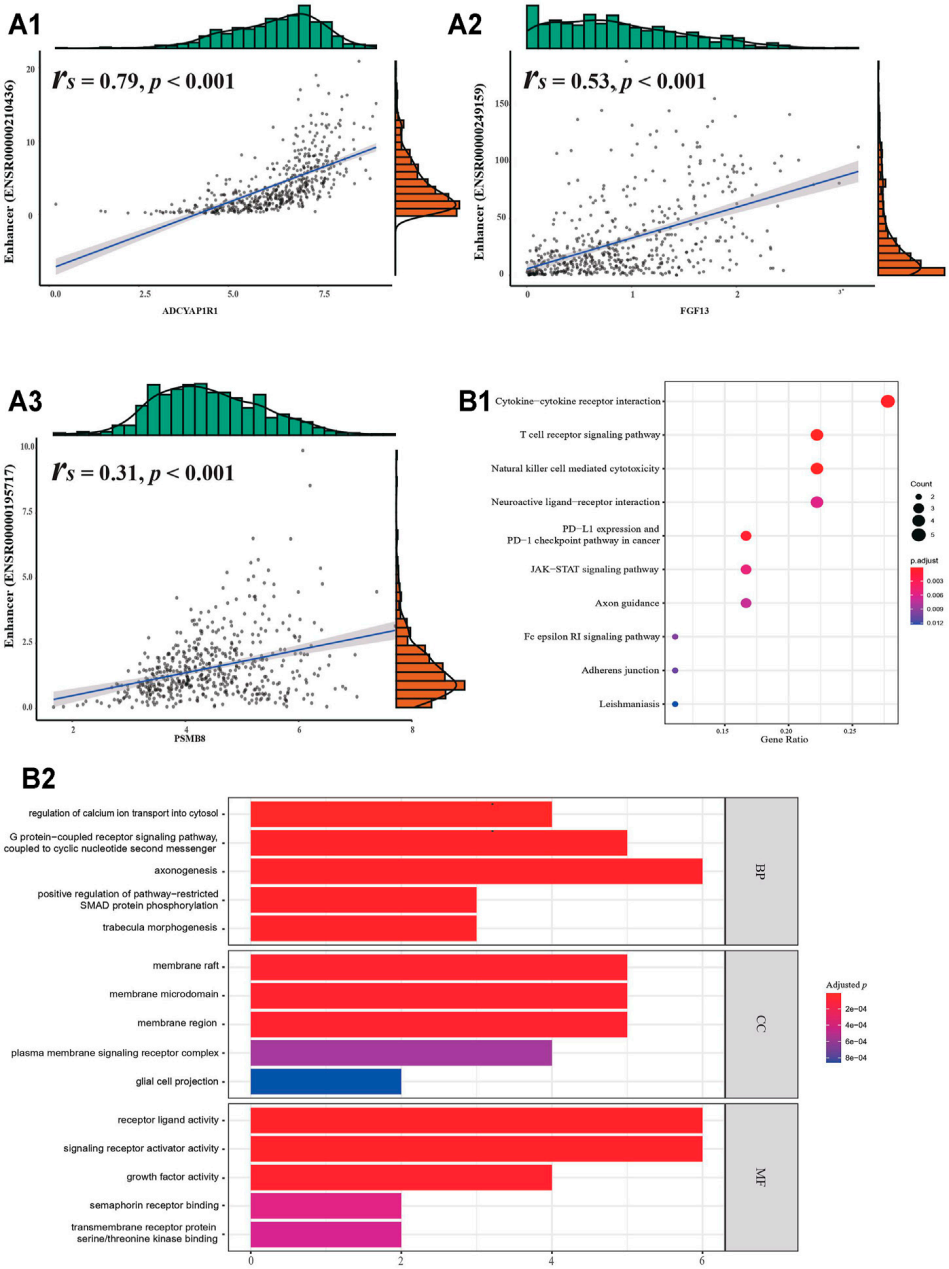
Identified target genes. **(A1)** Correlation between eRNA and its target gene. **(B1)** KEGG enrichment analysis. **(B2)** GO enrichment analysis.

Of the 461 eRNAs found to be expressed in gliomas, 274 affected survival (Zhang et al., 2019). Provided by previous research, these eRNAs regulated 1721 genes, of which 51 were immune-related (Zhang et al., 2019). To obtain a strong association between eRNAs and their target IRGs, 24 genes with ***r***_***s***_ < 0.3 were excluded, while 27 were included (Table 1; Figure 2A, and Supplementary Figure S1).

**TABLE 1:** The correlation analysis for eRNA and their target genes.

Gene type	Gene	eRNA	enhancer site	eRNA tissue	ERG	*r*_s_
specific-regulated	ADCYAP1R1	ENSR00000210436	7 (31027263)	gliomas	1 gene	0.787
specific-regulated	FGF13	ENSR00000249159	X (1,39720349)	gliomas	1 gene	0.531
specific-regulated	PSMB8	ENSR00000195717	6 (32867500)	gliomas	multi-genes	0.309
		ENSR00000195824	6 (33633665)	multi-tumors	multi-genes	0.254
seRNA-regulated	MAPT	ENSR00000094845	17 (44998305)	multi-tumors	multi-genes	0.480
		ENSR00000283518	17 (44999600)	multi-tumors	multi-genes	0.426
		ENSR00000094854	17 (45040640)	multi-tumors	multi-genes	0.265
seRNA-regulated	BMPR1A	ENSR00000031043	10 (87214200)	multi-tumors	multi-genes	0.408
		ENSR00000031044	10 (87216296)	multi-tumors	multi-genes	0.415
		ENSR00000260651	10 (87214700)	multi-tumors	multi-genes	0.411
seRNA- regulated	DDX17	ENSR00000146066	22 (37782552)	multi-tumors	multi-genes	0.562
		ENSR00000301859	22 (37781600)	multi-tumors	multi-genes	0.571
seRNA- regulated	ELN	ENSR00000213692	7 (74875000)	multi-tumors	multi-genes	0.366
		ENSR00000326719	7 (73284800)	multi-tumors	multi-genes	0.417
seRNA- regulated	BMP2	ENSR00000134110	20 (5817887)	multi-tumors	multi-genes	0.319
		ENSR00000134111	20 (5821900)	multi-tumors	multi-genes	0.325
		ENSR00000134112	20 (5823100)	multi-tumors	multi-genes	0.333
other	SEMA6C	ENSR00000013533	1 (150635378)	multi-tumors	multi-genes	0.428
		ENSR00000013524	1 (150596300)	multi-tumors	multi-genes	0.278
other	PDIA2	ENSR00000082228	16 (700964)	multi-tumors	multi-genes	0.360
other	PTPN6	ENSR00000048324	12 (6613700)	multi-tumors	multi-genes	0.419
other	SSTR5	ENSR00000082228	16 (700964)	multi-tumors	multi-genes	0.307
other	CD4	ENSR00000048324	12 (6613700)	multi-tumors	multi-genes	0.366

Functional enrichment analyses of the 27 target genes produced eight KEGG pathways and several GO terms (Figure 2B). The KEGG pathways were involved in the regulation of immune function, nervous function, and cell signaling. They included “PD-L1 expression and PD-1 checkpoint pathway in cancer”, “neuroactive ligand-receptor interaction”, and “JAK−STAT signaling pathway”. GO terms indicated that IRGs were enriched in various signaling pathways and “axonogenesis”, whose association with the TME has been reported previously (Han et al., 2021).

### Generation of a Prognostic Signature for Gliomas

Filtering through the LASSO Cox analysis, returned 13 IRGs (ADCYAP1R1, BMP2, BMPR1A, CD4, DDX17, ELN, FGF13, MAPT, PDIA2, PSMB8, PTPN6, SEMA6C, and SSTR5) significantly associated with survival outcomes. They were used to construct a comprehensive prognostic tool for gliomas. The risk scores for each patient were calculated using the parameters generated by the LASSO Cox model (Supplementary Figure S2 and Supplementary Table S5) applied to the following formula (Risk score = −0.16 ^∗^ ADCYAP1R1 − 0.37 ^∗^ BMP2 - 0.31 ^∗^ BMPR1A+ 0.03 ^∗^ CD4 − 0.01 ^∗^ DDX17 + 0.14 ^∗^ ELN − 0.11 ^∗^ FGF13 − 0.02 ^∗^ MAPT − 0.24 ^∗^ PDIA2 + 0.11 ^∗^ PSMB8 + 0.1 ^∗^ PTPN6 − 0.05 ^∗^ SEMA6C − 0.31 ^∗^ SSTR5).

**FIGURE 3:**
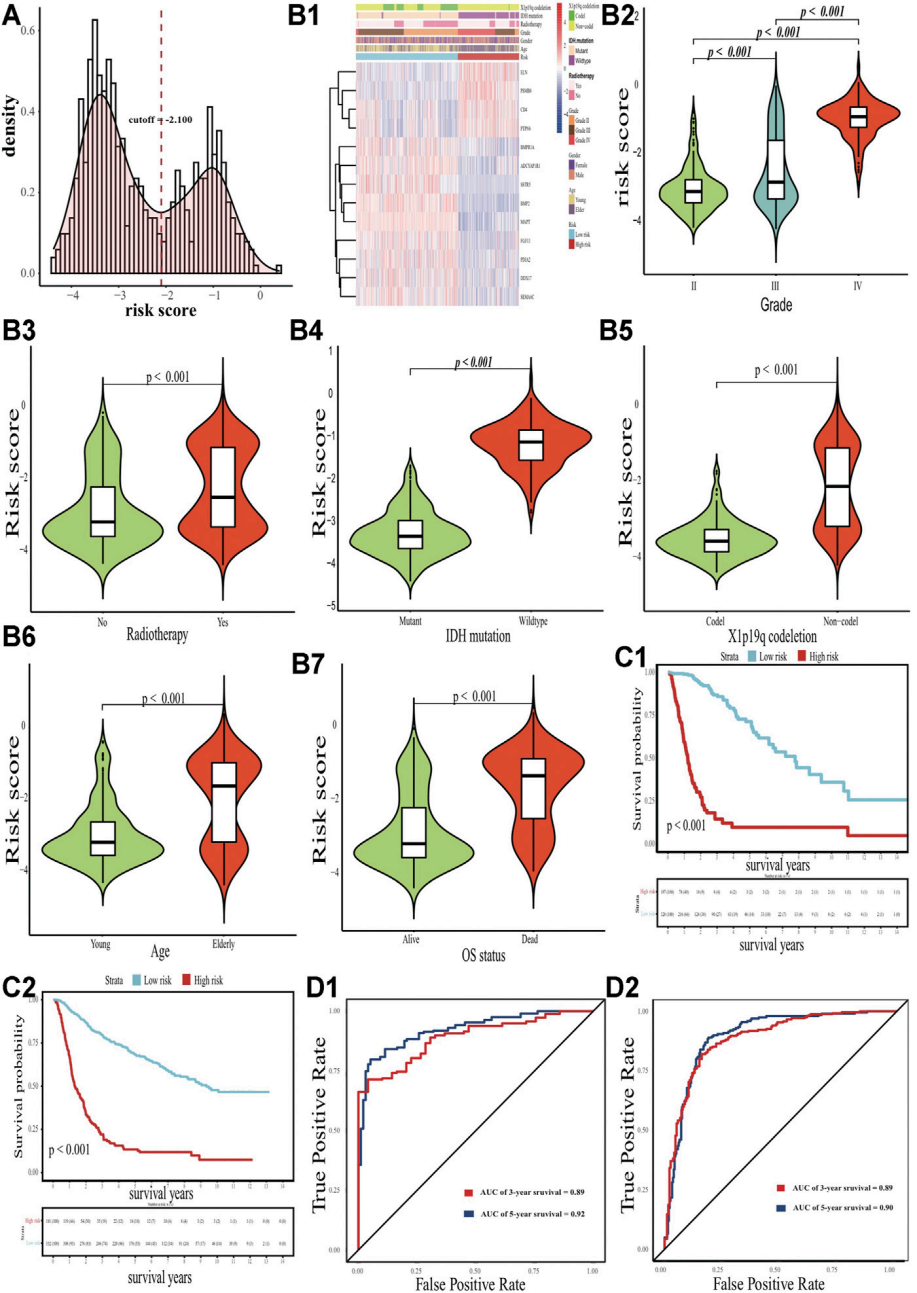
Clinicopathological differences with eRNA-related risk signature. **(A)** Distribution of the risk score. **(B)** Differences in expression profile, grade, radiotherapy status, IDH status, X1p19q status, age and survival status. **(C1-C2)** Kaplan-Meier curves for the TCGA and CGGA cohort, respectively. **(D1-D2)** ROC curves for the TCGA and CGGA cohort, respectively.

The distribution of the risk score pointed to two distinctive classes (328 patients in the low-risk group and 197 patients in the high-risk group) describing all glioma patients (Figure 3A). The two classes could be distinguished with respect to several clinicopathological features and gene expression profiles, including tumor grade, radiotherapy, IDH mutation status, X1p19q co-deletion status, age, and survival outcome (Figure 3B; *p* < 0.05). The Kaplan-Meier analysis demonstrated a clear difference in survival outcomes between the two classes, with low-risk patients surviving longer than their high-risk counterparts, regardless of development and validation cohorts (Figure 3C; *p* < 0.05). The AUCs of time-dependent ROC curves for the risk score were 0.91 and 0.88 in the TCGA cohort, and 0.86 and 0.84 in the CGGA cohort at 3 and 5 years, respectively (Figure 3D).

### Differences in the Immune Landscape Between Low- and High-Risk Patients

**FIGURE 4:**
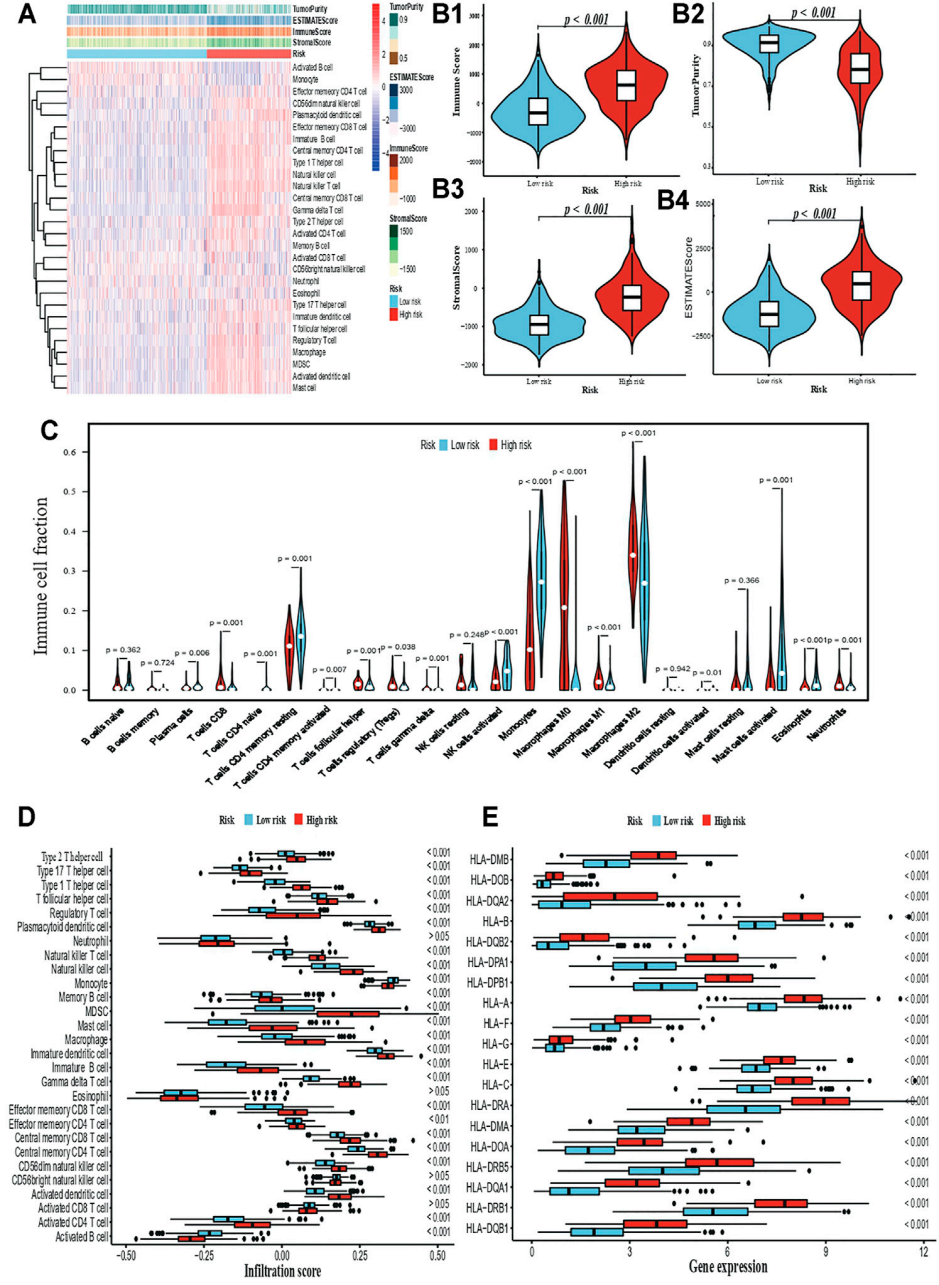
Immune-related comparison. **(A)** The ssGSEA profile. **(B1-B4)** Comparison for immune cell, tumor cell, and stromal cell in TME. **(C)** Infiltration comparison based on the ssGSEA algorithm. **(D)** Infiltration comparison based on the CIBERSORT algorithm. **(E)** Differential expression for HLA gene family.

Having established the glioma risk signature, we estimated the proportion of tumor, stromal, and immune cells in the TME of low- and high-risk patients (Figure 4A). In the latter, the TME exhibited heavier immune and stromal cell infiltration, but fewer tumor cells (Figure 4B; *p* < 0.05). The proportion of 22 immune cell types was calculated to determine the exact infiltration pattern between the two groups (Figure 4C; *p* < 0.05). In the low-risk group, activated NK cells, monocytes, and activated mast cells were the main types of immune cells; whereas CD8^+^ T cells, CD4^+^ memory resting T cells, M0 and M1 macrophages, eosinophils, and neutrophils were enriched in high-risk patients. Of note, only patients with *p* < 0.05 in the CIBERSORT estimation were used to compare immune infiltration (91 patients in the low-risk group and 59 patients in the high-risk group). Visible differences in immune infiltration levels were revealed by the ssGSEA method, with most immune cell types being more abundant in the high-risk group than in the low-risk group, except for monocytes and activated B cells (Figures 4A–D; *p* < 0.05). Except for the proportion of monocytes, the results in xCell were highly similar to those in CIBERSORT (Supplementary Figure S3A). Besides, the TIMER results also verified the high-risk patients suffered from a heavier immune infiltration burden in the local tumour patient’s microenvironment (Supplementary Figure S3B). In addition, 19 members of the HLA gene family were overexpressed in the high-risk groups (Figure 4E; *p* < 0.05).

### Predicting the Potential Effect of Immunotherapy and Chemotherapy

**FIGURE 5:**
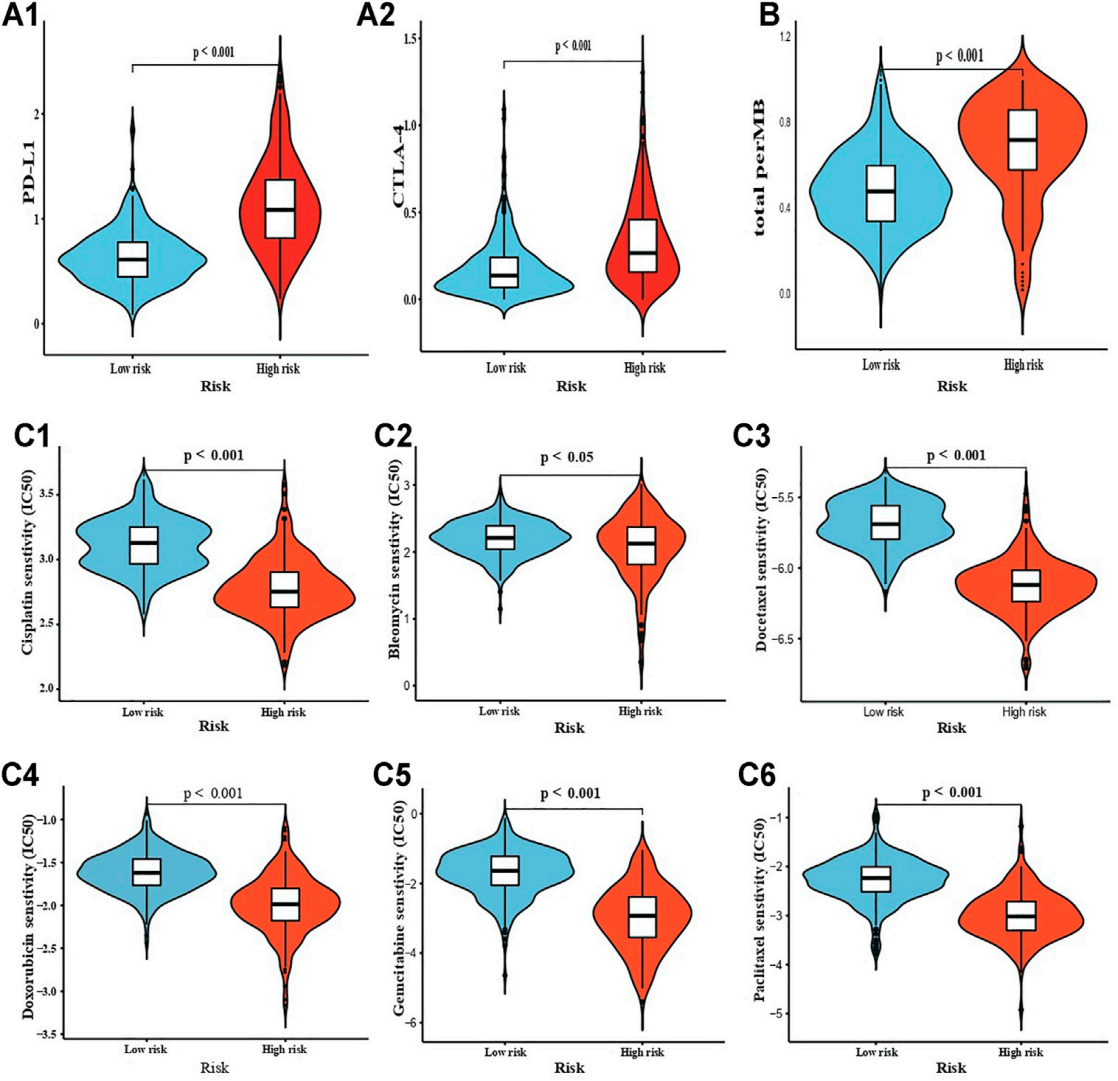
Drug sensitivity estimation. **(A1-A2)** Immunotherapy sensitivity estimation by PD-L1 and CTLA-4, respectively; and **(B)** TMB. **(C1-C6)** chemotherapy IC50 estimation for Cisplatin, Bleomycin, Docetaxel, Doxorubicin, Gemcitabine, and Paclitaxel.

PD-L1 and CTLA-4 were overexpressed in the high-risk group, indicating that these patients would have a higher response rate and more benefits from potential immunotherapy (Figure 5A; *p* < 0.05). Furthermore, the differential expression of PD-L1 and CTLA-4 were compared between lower-grade gliomas (LGGs; tumour grade II ∼ III) and glioblastoma in the high-risk group, and between different risk groups within the same tumour grade, such as PD-L1-grade IV-Low risk V.S. PD-L1-grade IV-High risk. There were no significant differential expressions of PD-L1 and CTLA-4 in the high-risk group (Supplementary Figures S4A, B; *p* > 0.05). However, most gene expression comparisons within the same tumour grade were significant (Supplementary Figures S4C–H; *p* < 0.05). Only the CTLA-4 expression in grade II or grade IV was insignificant, which may be attributed to the sample size of grade II in high-risk, and grade IV in low-risk. These analyses were to clarify whether the differential expression of PD-L1 and CTLA-4 was merely ascribed to differences between LGGs and GBM). To verify this from another point of view, the two groups were compared with respect to TMB. This comparison revealed that high-risk patients had a heavier TMB (Figure 5B; *p* < 0.001). IC_50_ prediction for six main common chemotherapeutics using the pRRophetic algorithm revealed that all high-risk patients had significantly lower IC_50_ (Figure 5C; *p* < 0.05). However, for temozolomide, there was insignificance. Taken together, these results indicated that patients in the high-risk group would benefit more from potential immunotherapy and chemotherapy.

### Variation in Metabolic Pathways

Pathway analysis revealed that signaling pathways participating in a variety of biological processes differed significantly between low- and high-risk patients (Supplementary Figure S5A). The high-risk group was associated preferentially with “WNT signaling pathways”, “taste transduction”, “MORT signaling pathway”, “long term depression”, “phosphatidylinositol signaling system”, and other 12 biological processes (Supplementary Figure S5B and Supplementary Table S6; |log2FC| > 0.3, adjusted *p* < 0.05). The low-risk group was associated primarily with “ECM receptor interaction”, “glutathione metabolism”, “glycosaminoglycan degradation”, “N-glycan biosynthesis”, “systemic lupus erythematosus”, and other 47 signaling pathways.

### Establishment and Validation of a Prognostic Model

**FIGURE 6:**
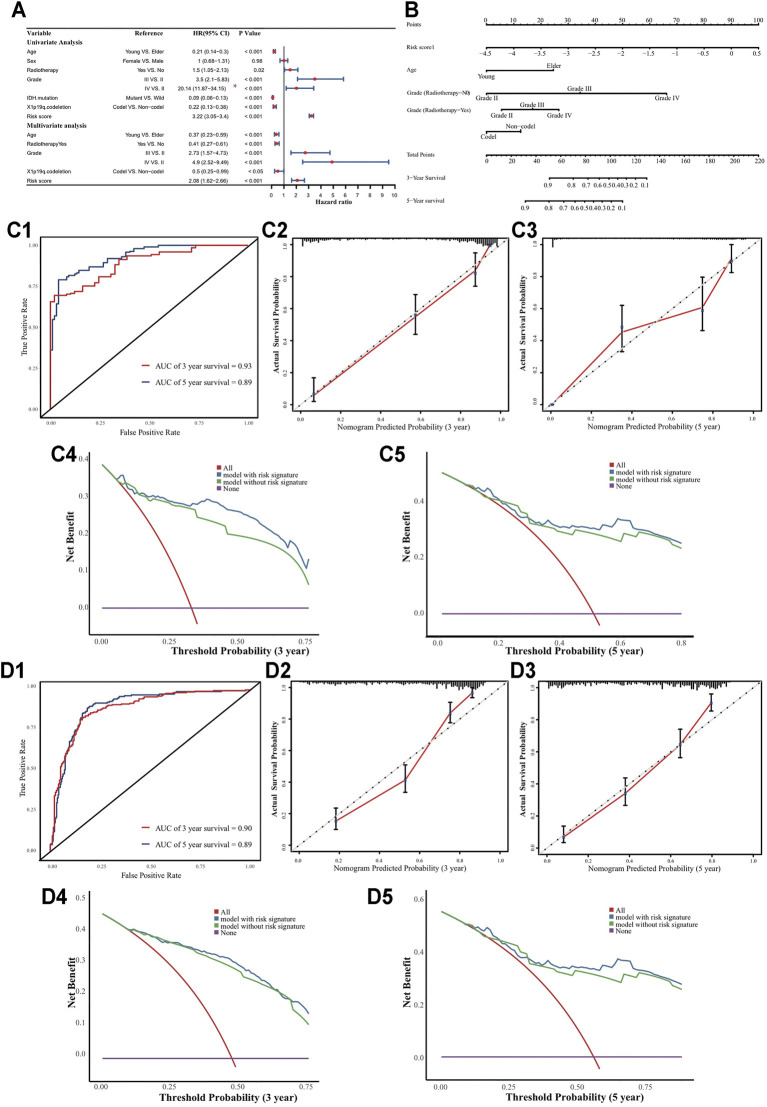
Nomogram to predict 3 and 5 years OS. **(A)** Univariate and multivariate analyses. The grade III HR and its CI were divided by 10 in the forest plot. **(B)** Nomogram to predict the 3-, 5- and 10-years OS for LGGs patients. **(C1)** Time-dependent ROC curves; **(C2-C3)** Calibration curves; **(C4-C5)** DCA curves in TCGA cohort. **(D1)** Time-dependent ROC curves; **(D2-D3)** Calibration curves; **(D4-D5)** DCA curves in CGGA cohort.

Based on univariate and multivariate Cox analyses for the risk score and the clinicopathological indicators mentioned above, the risk score, age, radiotherapy, tumor grade, and X1p19q co-deletion status were considered as independent indicators in a prognostic model (Figures 6A,B). A stepwise process was executed in the multivariate Cox model. However, these factors were not be used to establish a prognostic tool whose proportional hazards assumption was not be met, because of the radiotherapy status factor. To solve this problem, we introduced an interaction between radiotherapy and tumor grade into the established model, and its proportional hazards assumption was satisfied (Supplementary Figure S6; Schoenfeld test *p* = 0.16) (Wang et al., 2021a). Using 1,000 bootstrap iterations, the concordance indices of the established model were calculated as 0.87 (95% CI: 0.84–0.90) in the TCGA cohort and 0.80 (95% CI: 0.78–0.83) in the CGGA cohort. Actual discriminative performance was assessed by ROC curves at 3 and 5 years (AUCs: 0.93 and 0.89 in the TCGA cohort; 0.90 and 0.89 in the CGGA cohort) (Figures 6C,D). The robustness of the model’s predictive ability was evaluated by the calibration curves and its clinical utility by decision curve analysis in the TCGA and CGGA cohorts (Figures 6C,D). Besides, we used our established prognostic model to LGGs alone, to verify the robustness and efficiency were not merely resulted from the differences between LGGs and GBM. Similar performances were observed: Harrell’s concordance index 0.85 (95% CI: 0.80–0.89); AUCs 0.87 and 0.79 at 3 and 5 years in the development cohort, 0.88 and 0.85 at 3 and 5 years in the validation cohort; robust calibration curves (Supplementary Figure S7; *p* < 0.001).

**FIGURE 7:**
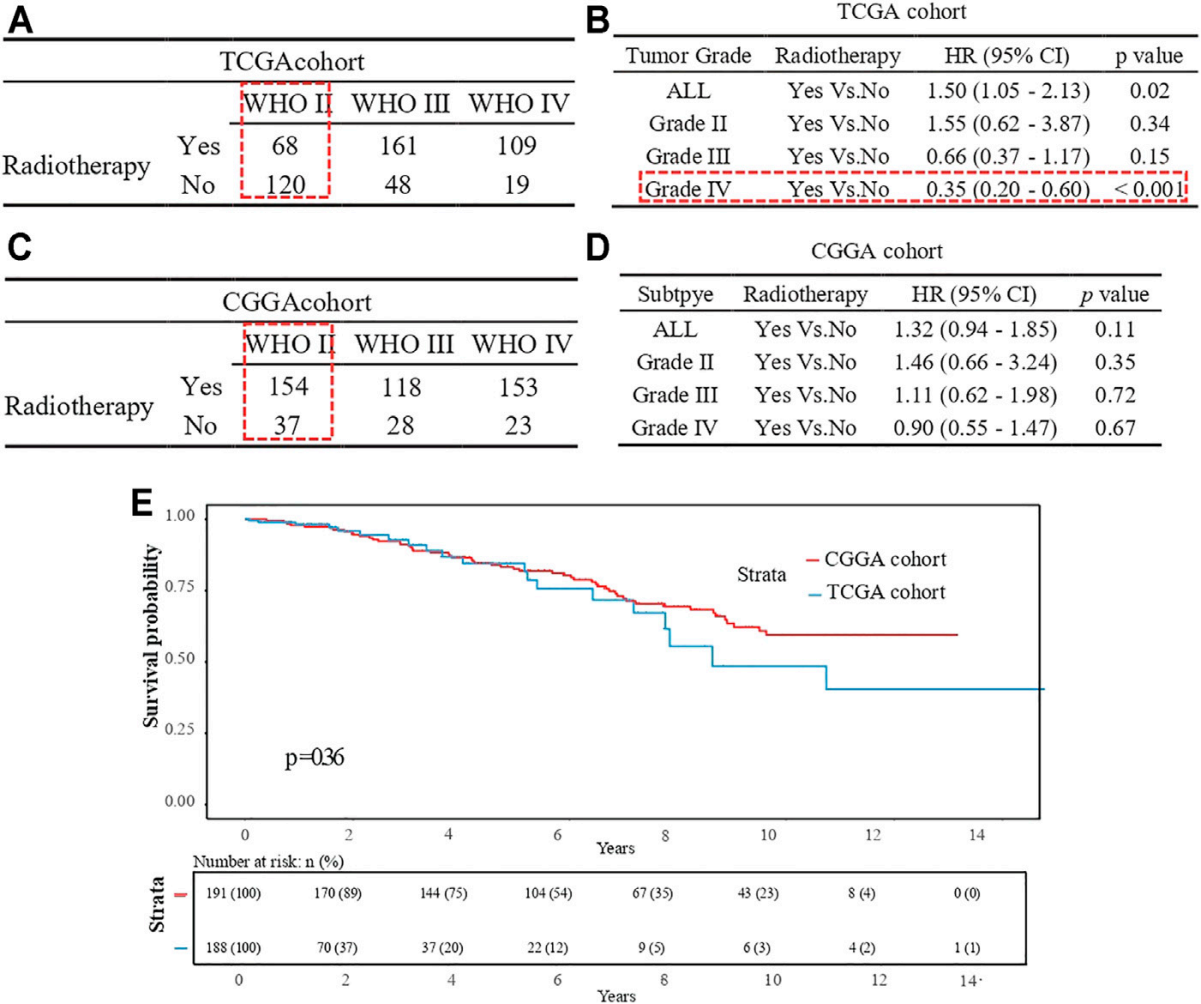
Radiotherapy status comparison for grade III patients. **(A)** Frequency table in TCGA cohort. **(B)** Stratification analysis in TCGA cohort. **(C)** Frequency table in CGGA cohort. **(D)** Stratification analysis in CGGA cohort. **(E)** Kaplan-Meier curve for the two cohort patients with tumor grade II.

Interestingly, the prognostic effect of radiotherapy status contrasted between the univariate and multivariate Cox models. We hypothesized that the discrepancy might be due to tumor grade acting as an important confounding factor. Indeed, the radiotherapy rate was higher for grade III-IV patients than grade II patients in the TCGA cohort (Figure 7A; *p* < 0.05). Stratification analysis confirmed that tumor grade was a confounding factor, and radiotherapy was a protective factor for grade IV patients (Figures 7B–D; HR = 0.35, *p* < 0.05). Furthermore, we found that the radiotherapy rate for grade II patients was higher in the CGGA cohort than in the TCGA cohort (Figure 7C; *p* < 0.05), and verified that radiotherapy had no obvious prognostic value for them (Figure 7E; *p* = 0.36).

### Comparison of Genomic Alterations

Chr 7+/10- was found to occur more frequently with a higher risk signature (Supplementary Figure S8A; *p* < 0.001). Patients with lower-risk signatures had higher MGMT methylation levels (Supplementary Figure S8B; *p* < 0.001). A comparison of TERT gene expression indicated that patients with higher risk signatures were more likely to have accumulated TERTp mutations (Supplementary Figure S8C; *p* < 0.001).

### Difference Between Subtype Grade Groups

We found that grade III patients from both TCGA and CGGA cohorts accounted for a large portion of both the high- and low-risk groups, whereas the same was not observed for grade II and IV patients (Figure 3B and Supplementary Figure S9). This finding suggested the existence of potential subgroups within-grade III patients, which we defined as high-risk grade III (H-III) and low-risk grade III (L-III).

**FIGURE 8:**
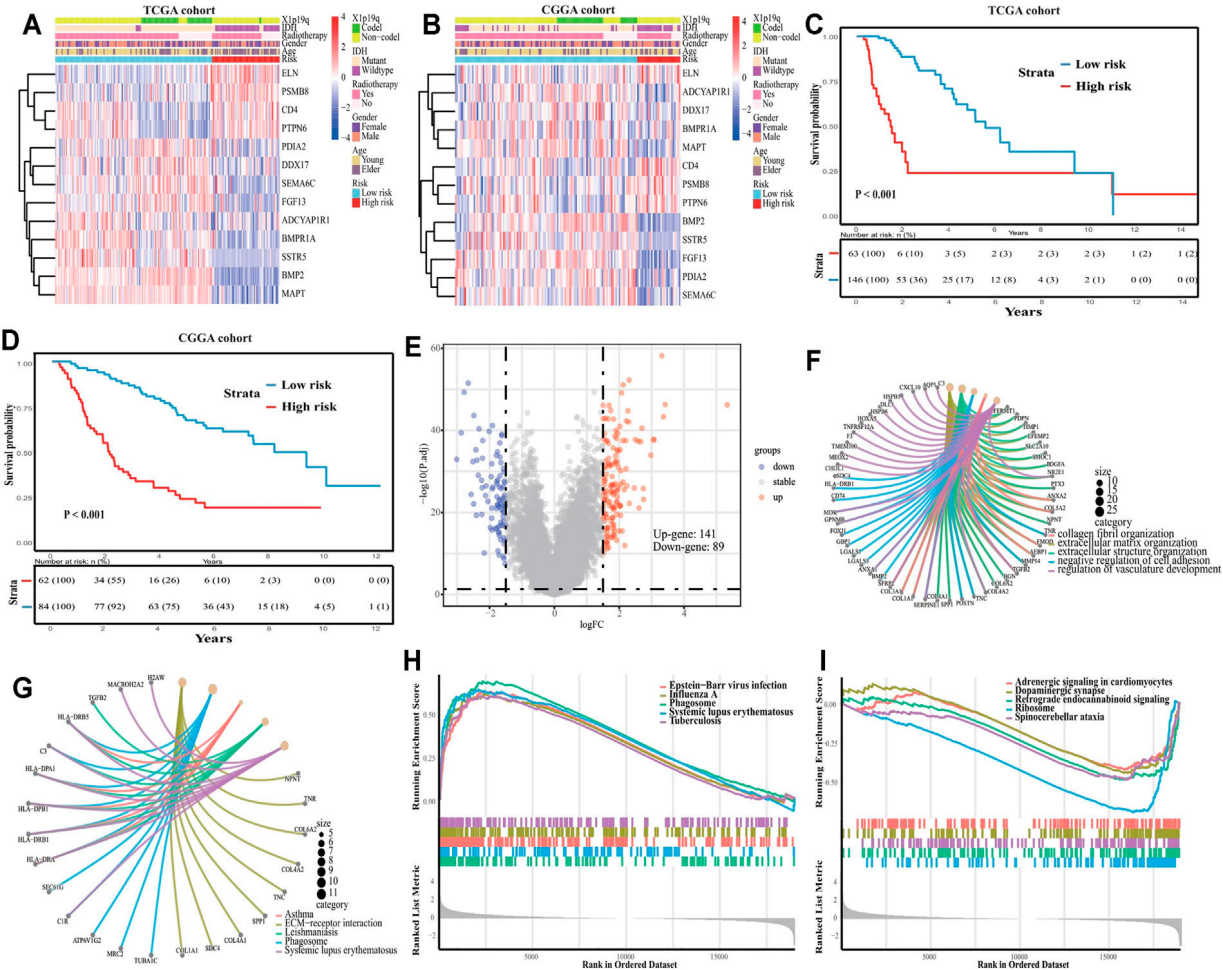
Subclass analysis for tumor grade III. **(A)** Gene expression profile in TCGA cohort, and **(B)** in CGGA cohort. **(C)** Kaplan-Meier curve for TCGA cohort patients, and **(D)** for CGGA cohort patients. **(E)** Volcano plot between high- and low-risk groups. **(F)** GO enrichment for differential genes**. (G)** KEGG enrichment for differential genes**. (H)** GSEA in the high-risk score, and **(I)** in low-risk score.

L-III and H-III patients displayed clear differences in gene expression profiles, as well as survival outcomes (Figures 8A–D; *p* < 0.05). A total of 230 differentially expressed genes were identified between the two groups, including 141 upregulated genes and 89 downregulated genes (Figure 8E; |Log2FC| > 1.5; adjusted *p* < 0.05). GO analysis revealed significant enrichment for “collagen fibril organization”, “extracellular matrix organization”, “extracellular structure organization’, “negative regulation of cell adhesion”, and “regulation of vasculature development’ pathways (Figure 8F; *p* < 0.05). At the same time, “allograft rejection”, “asthma”, “ECM-receptor interaction”, “leishmaniasis”, and “phagosome” pathways were significantly enriched following KEGG analysis (Figure 8G; *p* < 0.05). To identify potential signaling pathways explaining the discrepancy between the two subtypes, GSEA was used to identify significant KEGG pathways based on the results of differentially expressed genes. The top five signaling pathways for the H-III group were “Epstein−Barr virus infection”, “focal adhesion”, “human T-cell leukemia virus 1 infection”, “influenza A”, and “lysosome” (Figure 8H; enrichment score >0.3, *p* < 0.05); whereas for the L-III group, the activated pathways were ‘cAMP signaling pathway”, “coronavirus disease-COVID-19”, “dopaminergic synapse” “neuroactive ligand-receptor interaction”, and “olfactory transduction” (Figure 8I; enrichment score < −0.3, *p* < 0.05). Many of these signaling pathways are involved in tumor immune functions and HLA malfunction, which confirms the need to explore potential mechanisms underlying the diverse subtypes defined by eRNAs and their target genes.

### Contribution of eRNA in Target Gene Expression Variance

The main CNVs were concentrated in some most studied genes, such as TP53, IDH1, ATRX, TTN, PTEN (Supplementary Figure S10A). However, for IRGs, the CNVs were too low to influence the gene expression (Supplementary Figure S10B; mutation rate <1%). Therefore, the CNVs were excluded from the multivariate model. Besides, it was obvious that the eRNAs were important factors to influence the target expression (Table 2). Though eRNAs and methylation were all crucial, eRNAs were more important factors to influence the IRGs expression. The eRNA even could explain 70.83% of the known variance for the DDX17 gene. These could indicate that eRNAs occupied an important position and were worth studying in the gene regulation system.

**TABLE 2:** The contribution of eRNA in target gene expression variance.

Gene	Coef-M	*p*	Coef-e	*p*	Con_M	Con_e	Con_T	Pro_M	Pro_e
ADCYAP1R1	1.08	0.14	1.80	<0.01	<0.01	0.26	0.51	<1.00%	50.98%
FGF13	—	—	1.45	<0.01	-	0.12	0.23	-	38.24%
PSMB8	0.84	<0.01	1.72	<0.01	0.03	0.28	0.54	5.56%	51.85%
MAPT	1.00	0.98	1.09	<0.05	<0.01	<0.01	0.53	<1.00%	<1.00%
BMPR1A	0.79	<0.01	0.82	0.24	0.04	0.04	0.38	10.53%	10.53%
DDX17	0.92	0.18	33.88	<0.01	<0.01	0.34	0.48	<1.00%	70.83%
ELN	0.73	<0.01	1.16	<0.05	0.06	0.07	0.38	15.79%	18.42%
BMP2	0.66	<0.01	1.13	0.34	0.07	0.02	0.77	9.09%	2.60%
SEMA6C	0.73	<0.01	1.20	<0.01	0.09	0.06	0.39	23.08%	15.38%
PDIA2	1.00	1.00	1.21	<0.01	<0.01	0.03	0.21	<1.00%	14.29%
PTPN6	0.71	<0.01	1.55	<0.01	0.13	0.15	0.60	21.67%	25.00%
SSTR5	—	—	1.11	<0.01	—	0.01	0.37	—	2.70%
CD4	0.81	<0.01	1.53	<0.01	0.04	0.14	0.47	8.51%	29.79%

The table results were all collected from the multivariable model, including age, grade, gender, radiotherapy, IDH, mutation, 1p19q codeletion, target gene methylation level, and eRNA, level. Coef-M: coefficient of methylation; Coef-e: coefficient of eRNA; Con_M: contribution of methylation in the explained sum of squares (ESS); Con_e: contribution of eRNA, in the ESS; Con_T: total ESS; Pro_M: the ratio of Con_M in Con_T; Pro_e: the ratio of Con_e in Con_T. Please note the methylation level of FGF13 and SSTR5 was not be detected, so relevant statistics were ignored.

### Knockdown of PTPN6 and PSMB8 and Their eRNAs

**FIGURE 9:**
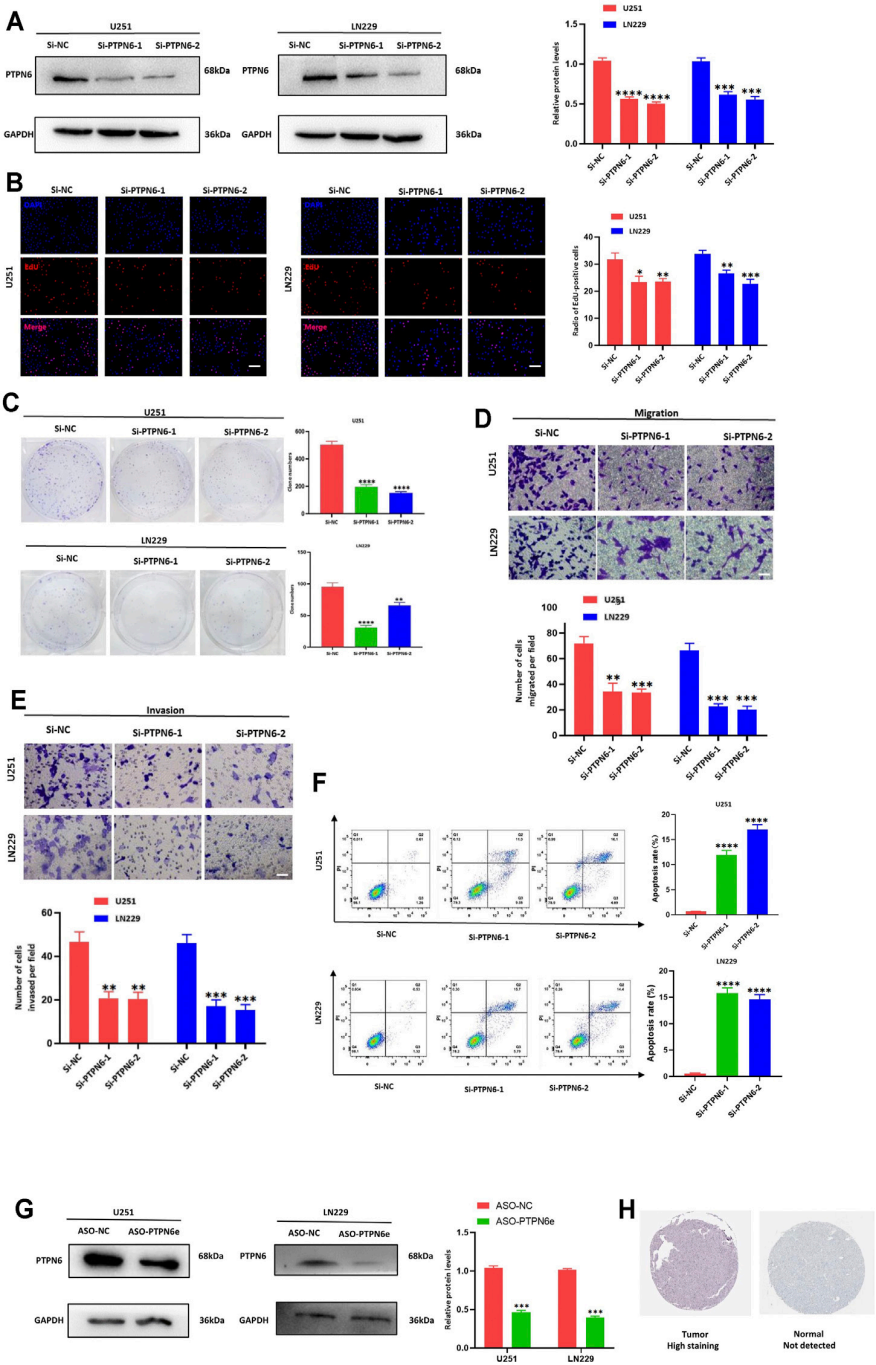
Knockdown of PTPN6 suppressed the proliferation, migration, invasion, and increased cell apoptosis of U251 and LN229 cells *in vitro*. **(A)** Western blot analysis to examine the efficiency of the PTPN6 knockdown. **(B)** EdU staining to assess proliferation ability. **(C)** Clonogenic assays to assess colony-forming abilities. **(D,E)** Transwell assays to detect the migration and invasive capacities. **(F)** Flow cytometry to analyze the apoptosis of U251 and LN229 cells. **(G)** Relative quantitation of PTPN6 protein expression levels in ASO-treated U251 and LN229 cells. **(H)** PTPN6 expression in glioma tissues and normal tissues. Magnification, ×200 **(B,D, and E)**. Scale bar, 100 μm **(B,D, and E)**. *p < 0.05; **p < 0.001; ***p < 0.001; ****p < 0.0001. Data are shown as mean ± SD at least three independent experiments.

The si-PTPN6 and si-PSMB8 targeting PTPN6 and PSMB8 were transfected into the U251 and LN229 cells. According to the western blot analysis, both of the two selected siRNAs could significantly decrease PTPN6 and PSMB8 expression as shown in both cell types (Figure 9A and Supplementary Figure S11A; *p* < 0.001). Next, EdU staining and colony formation assays were performed to assess the cell proliferation. The results indicated that compared with the control (si-NC), the silence of PTPN6 and PSMB8 significantly suppressed cell growth (Figure 9B and Supplementary Figure S11B; *p* < 0.05), and the formation of tumor cell colonies (Figure 9C and Supplementary Figure S11C; *p* < 0.01).

We further explored the potential impact of PTPN6 and PSMB8 on migration and invasion by transwell assays. U251 and LN229 cells transfected either with si-PTPN6 and si-PSMB8 presented a dramatically inhibited migration and invasion ability (Figures 9D,E and Supplementary Figures S11D, E; *p* < 0.01). Consistently, the apoptosis rates were both higher in the si-PTPN6 and si-PSMB8 silence group than in the negative control (Figure 9F and Supplementary Figure S11F; *p* < 0.0001). Those results suggested a critical role of PTPN6 and PSMB8 in the cell proliferation and aggressiveness of glioma cells. Knockdown of eRNA by ASO could significantly suppress target gene expression (Figure 9G and Supplementary Figure S11G; *p* < 0.001). Besides, the IHC confirmed that high levels of PTPN6 and PSMB8 expression occurred in glioma tissues (Figure 9H and Supplementary Figure S11H).

## Discussion

So far, it has remained unclear how IRGs alter the TME and how this impacts survival outcomes. Numerous studies have demonstrated that the TME and immune-related characteristics are closely linked to disease prognosis, as indicated by a faster tumor progression with increasing invasiveness of tumor cells (Wang et al., 2017; Ma et al., 2018). In this context, it is important to identify which gene is dysfunctional and what is the cause of its malfunction. As pivotal regulatory biomarkers, eRNAs have various tumor-specific features such as the regulation of key immune checkpoints (Zhang et al., 2019; Lee et al., 2020). In this study, eRNA-regulated IRGs identified in glioma patients were used to construct a comprehensive and highly robust risk signature capable of predicting individual outcomes. Based on the distribution of IRGs in the risk signature, patients were divided into low- and high-risk groups. The characteristics of their TME were compared to reveal potential mechanisms explaining the heterogeneity in OS. Besides, we had found that for IRGs, the eRNAs may make an important contribution to gene regulation. Notably, the research approach used in this study has great potential for identifying eRNA-regulated oncogenes and other types of malignant cancers.

The TME of low-risk patients was significantly more pure, meaning that tumor cells recruited fewer non-tumor cells, formed a stable solid tumor, and became less invasive (Zhang et al., 2017). In contrast, the high-risk phenotype was enriched in non-tumor cells, such as macrophages and neutrophils, leading to greater immune infiltration and worse survival outcomes. A hyperactive immune response in local tumor tissues blocks attacks from beneficial immune cells, such as NK cells and CD4^+^ T cells (Cheng et al., 2016; Zhang et al., 2017). Although similar findings have been reported in many types of malignant tumors, such as gliomas and colorectal carcinoma, the underlying mechanism remains largely unknown (Cheng et al., 2016; Zhang et al., 2017; Mao et al., 2018). Upon comparing immune infiltration levels, it was found that most immune cells were much more abundant in tumor tissues of high-risk patients. Contradictory results on immune infiltration obtained by ssGSEA and CIBERSORT might be ascribed to a smaller sample size upon CIBERSORT analysis. Nevertheless, the discrepancy in immune infiltration between the two risk groups was very clear. In this study, macrophages were extremely abundant in the local TME of high-risk patients. Their recruitment contributes to immune escape and immune resistance. In particular, macrophage subtypes M1 and M2 have been related to tumor growth and invasion (Hambardzumyan et al., 2016). A comparison of the TME between patient subtypes points to a link between immune dysfunction and eRNA malfunction, which singularly regulates IRGs, affects crucial biological pathways and, ultimately, alters OS.

It is difficult to make an informed clinical decision about the most beneficial course of therapy for malignant tumors, especially refractory malignant gliomas. The differences identified here in the TME highlight the potential benefits of immunotherapy for glioma patients, particularly if aimed at immune checkpoint inhibitors. Checkpoint expression levels and TMB are primary indicators of the feasibility of immunotherapy (Chalmers et al., 2017). A heavier TMB could be related to mismatch repair defects, which could be treated with drugs targeting specifically PD-1/PD-L1 (Le et al., 2017). Moreover, a higher expression of checkpoint genes, such as PD-L1 and CTLA-4, in these patients could improve the efficacy of immunotherapy. We not only found that high-risk patients would benefit more from potential immunotherapy, but we also show that this difference derives in part from the malfunction of eRNAs. Though our study found the differences of potential immunotherapy effects and verify it was not merely attributed to the differences between LGGs and GBM, we must emphasize that up to now, the failures of PD-1/PD-L1 immunotherapy for glioma had been verified in phase III clinical trial (Filley et al., 2017; Zhao et al., 2019a; Reardon et al., 2020). However, researchers have proven that the tumour microenvironment has enough immune cell infiltration to activate the immune response (Louveau et al., 2015; Wang et al., 2021b). Besides, *in vitro* experiment, the PD-1/PD-L1 or CTLA-4 blockade have got suppression effect (Zhao et al., 2019b). It reminded us that immunotherapy might benefit glioma patients, but more effective immune targets were needed (Wang et al., 2021a). Even though the higher sensitivity of high-risk patients to common chemotherapeutic agents (e.g., temozolomide) could not be found, it is reasonable to assume that a diverse drug susceptibility applies to different risk patients. This study anticipates the differences in efficacy for potential chemo/immunotherapy, which could inform clinicians on the most rational decisions in clinical practice.

The present study revealed significant variations in the biological pathways enriched in low-as opposed to high-risk patients. Many complex pathways were identified by GSVA, offering a broad spectrum of candidate genes involved in tumorigenesis, progression, and therapeutic outcomes of gliomas. Specifically, the different pathways were found to be related mainly to cellular signal transduction, DNA replication and damage repair, as well as metabolism. Many of them have been proven to be inextricably linked to tumorigenesis, cell proliferation, immune cell differentiation, and survival outcomes (Zou et al., 2020; Colardo et al., 2021; Lou et al., 2021). Pathway enrichment (GO and KEGG) analysis for survival-related genes returned several pathways already found by GSVA, further confirming their vital role in controlling the biology of gliomas. Enrichment results support the involvement of survival-mediating eRNA-targeted genes in biological processes directly regulating the nervous system, such as “axonogenesis”, “glial cell projection”, “neuroactive ligand-receptor interaction”, and “axon guidance’.

The most prominent contribution of this study is the identification of eRNA-regulated IRGs. Among the identified genes, eight were controlled specifically by eRNA or seRNA. This finding confirmed how specific eRNAs and seRNAs were important factors affecting survival outcomes. Glioma-specific eRNAs and their downstream genes have great potential as therapeutic targets. One of the reasons preventing effective treatment of gliomas is their huge heterogeneity, which makes it difficult to formulate treatment and management plans. Glioma-specific eRNAs provide novel opportunities to overcome this problem. For example, the eRNA of ENSR00000210436 located on chromosome 7 (from 31024263 to 31030263) was only expressed in gliomas and was strongly correlated with its target gene (*r*_*s*_ = 0.79, adjusted *p* < 0.05) (Zhang et al., 2019). In addition, strong correlations were observed among seRNAs, further supporting their identity as super-enhancer RNAs not only because they were localized to adjacent sites, but also because they were co-expressed in tumor tissues.

In terms of potential clinical applications, this study used the identified genes to construct an innovative prognostic model and assess its robustness, specificity, and clinical utility. The model’s performance showed elevated reliability. Quantitative indicators of discrimination capacity (concordance index and AUCs) were higher than in many previous studies (Zhang et al., 2017; Qian et al., 2018), which may be related to the specificity and effectiveness of eRNAs. Moreover, the risk signature was closely related to genomic alterations, which reinforces the notion that abnormally activated target genes do not act alone but crosstalk with other important factors affecting survival outcomes, including the TERTp mutation, MGMTp methylation, and chr 7+/10- status. Previous studies have reported a link between these genomic alterations and survival outcomes, but the present results facilitate further investigation of the mechanisms underlying tumor progression (Brat et al., 2018; Galbraith et al., 2020; Heravi Shargh et al., 2021). Furthermore, we found that radiotherapy failed to promote survival outcomes in grade II patients, likely increasing only their financial and social burden.

Based on the gene expression profile, diverse subtypes of grade III patients were detected in the TCGA cohort and verified in the independent CGGA cohort. Tumor grade II or IV patients were not explored for additional subtypes because these accounted only for tiny percentages in the TCGA or CGGA cohorts and, thus, could not provide robust proof. GSEA was performed to identify relevant KEGG pathways, with which to explore the potential mechanisms explaining grade III subtypes. Immune-related signaling pathways were enriched in the H-III group, indicating that an overactive local immune response was an important pathogenic determinant, which led to more serious outcomes. For example, HLA-related pathways were singularly activated, which may be an important determinant causing biological differences, and has been proven to be related to clinicopathological features and survival outcomes of glioma patients (Wu et al., 2020).

In our study, the risk score was highly related to IDH status in the TCGA cohort. Although the observed relationship was weaker in the CGGA cohort, this fact supports the need to exploit immune-related and survival-related mechanisms as contributors to gliomas. A similar phenomenon was observed with respect to X1p19q co-deletion status, explaining why only the latter and not IDH status was included in the prognosis for gliomas. The exact roles of IDH status and X1p19q co-deletion status in gliomas could be elucidated by future work on eRNAs, enhancers, or their target genes.

The tumor suppressor protein tyrosine phosphatase non-receptor type 6 (PTPN6 or SHP1) is a tyrosine phosphatase involved in the regulation of numerous intracellular signaling cascades that control cell proliferation, differentiation, and apoptosis. Prior studies had found that the dysregulation of its expression can cause abnormal cell growth and promote tumor formation in leukemia (Wu et al., 2003). Proteasome subunit beta type-8 (PSMB8) is a member of the 20S proteasome (Kimura et al., 2015). The overexpression of PSMB8 contributes to the progression of gastric cancer, which is related to the degree of gastric cancer differentiation, the depth of tumor invasion, lymph node metastasis and depth of invasion (Kwon et al., 2016). According to a series of bioinformatics analyses and experiments, we found that silencing PTPN6 and PSMB8 in U251 and LN229 cells, significantly decreased cell viability, clone formation, migration and invasion ability, and induced cell apoptosis and the eRNAs were important factors to regulate the gene expression.

The present report has also some limitations, which should be addressed in future studies. First, more reliable evidence from prospective studies should be provided prior to translation into clinical practice. Second, the established model contains many genetic determinants, which aggravates the economic burden on patients and diminishes its usefulness. Third, the locations of enhancers on chromosomes and the direct regulatory relationships between eRNAs and target genes should be confirmed in rigorous experiments. Fourth, only an eRNA-regulated gene was robustly validated in our study, and the rest should be accomplished similar validation in the future study, especially for the specific-RNA/seRNA-regulated genes. Finally, multi-omics data should be integrated to generate a more complete biological regulatory network and reveal additional information, such as long non-coding RNAs, micro RNAs, and epigenetic mutations.

In conclusion, this study identified 13 eRNA-regulated IRGs and integrated them in a comprehensive signature to serve as an accurate prognostic factor. The risk signature offered a good discriminative ability towards TME characteristics, and patients with different risk levels displayed significantly different sensitivities to chemo/immunotherapeutic agents. The established model could be used to predict survival outcomes with high specificity and robustness, and aid clinicians in making more rational decisions. Furthermore, specific eRNAs and their regulated genes could be explored as therapeutic targets for refractory tumors, as well as cancers other than gliomas.

## Data Availability

The datasets presented in this study can be found in online repositories. The names of the repository/repositories and accession number(s) can be found in the article/Supplementary Material.

## Supplementary Material

Supplementary Figure S1 Correlation between eRNA and its target gene.

Supplementary Figure S2 Lasso Cox result graph. **(A)** The selection of lambda penalty parameter for the Lasso-Cox model. **(B)** The coefficient change curve of variables.

Supplementary Figure S3 Estimation of local immune infiltration. **(A)** xCell estimation; **(B)** TIMER estimation.

Supplementary Figure S4 PD-L1 and CTLA-4 comparison. **(A-D)** comparison between LGGs and GBM; **(E-F)** comparison between high-risk and low-risk patients with tumour grade III; **(G-H)** comparison between high-risk and low-risk patients with GBM.

Supplementary Figure S5 GSVA analysis. **(A)** The heatmap of GSVA result. **(B)** Differential pathways.

Supplementary Figure S6 Proportional hazards test figure.

Supplementary Figure S7 Nomogram in subclass patients. **(A-D)** The calibration, ROC curves, and Kaplan-Meier curve in the TCGA cohort. **(E-F)** The calibration, ROC curves, and Kaplan-Meier curve in the CGGA cohort.

Supplementary Figure S8 The comparison of genomic alterations. **(A)** Chr 7+/10-; **(B)** MGMT methylation; and **(C)** TERT gene expression.

Supplementary Figure S9 The gene expression profile of the CGGA cohort.

Supplementary Figure S10 the CNAs of the target gene. **(A)** Top 20 CNA genes; **(B)** The CNAs of target genes.

Supplementary Figure S11 Knockdown of PSMB8 suppressed the proliferation, migration, invasion, and increased cell apoptosis of U251 and LN229 cells in vitro. **(A)** Western blot analysis to examine the efficiency of the PSMB8 knockdown. **(B)** EdU staining to assess proliferation ability. **(C)** Clonogenic assays to assess colony-forming abilities. **(D and E)** Transwell assays to detect the migration and invasive capacities. **(F)** Flow cytometry to analyze the apoptosis of U251 and LN229 cells. **(G)** Relative quantitation of PSMB8 protein expression levels in ASO-treated U251 and LN229 cells. **(H)** PSMB8 expression in glioma tissues and normal tissues. Magnification, × 200 **(B, D, and E)**. Scale bar, 100 μm **(B, D, and E)**. * *p* < 0.05; ** *p* < 0.001; *** *p* < 0.001; **** *p* < 0.0001. Data are shown as mean ± SD at least three independent experiments.
