# Supplementary figures and images for: A Novel Prognostic Tool for Glioma Based on Enhancer RNA-Regulated Immune Genes
Source: Front Cell Dev Biol. 2022 Jan 20;9:798445. doi: 10.3389/fcell.2021.798445 (PMC8811171; doi:10.3389/fcell.2021.798445)

A

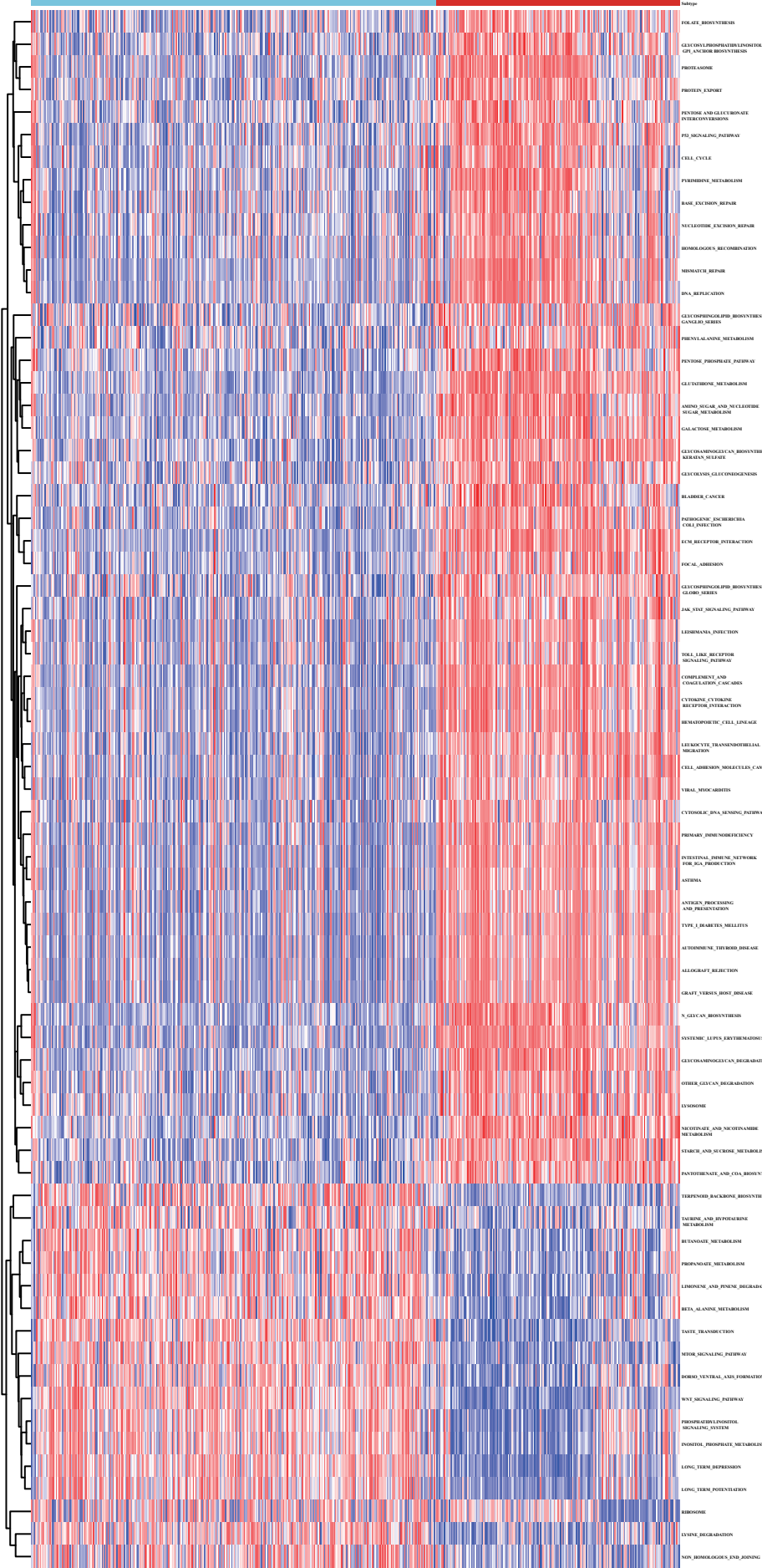

B

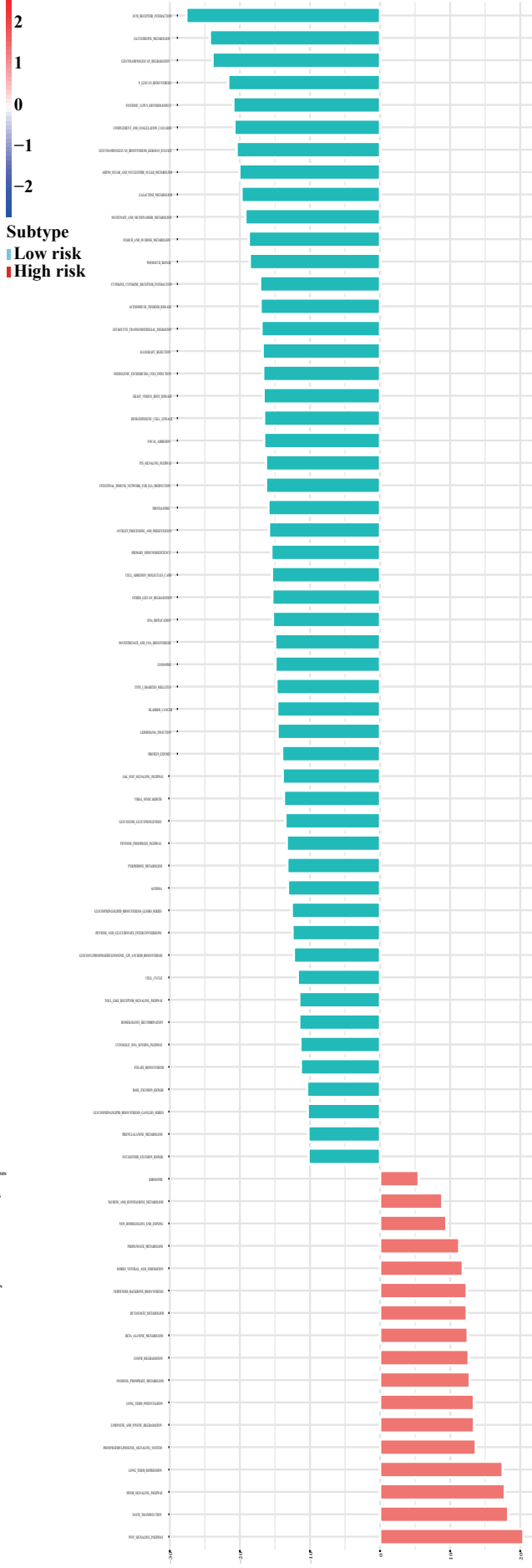

Supplement: Supplementary file 1 [file Image5.PDF]

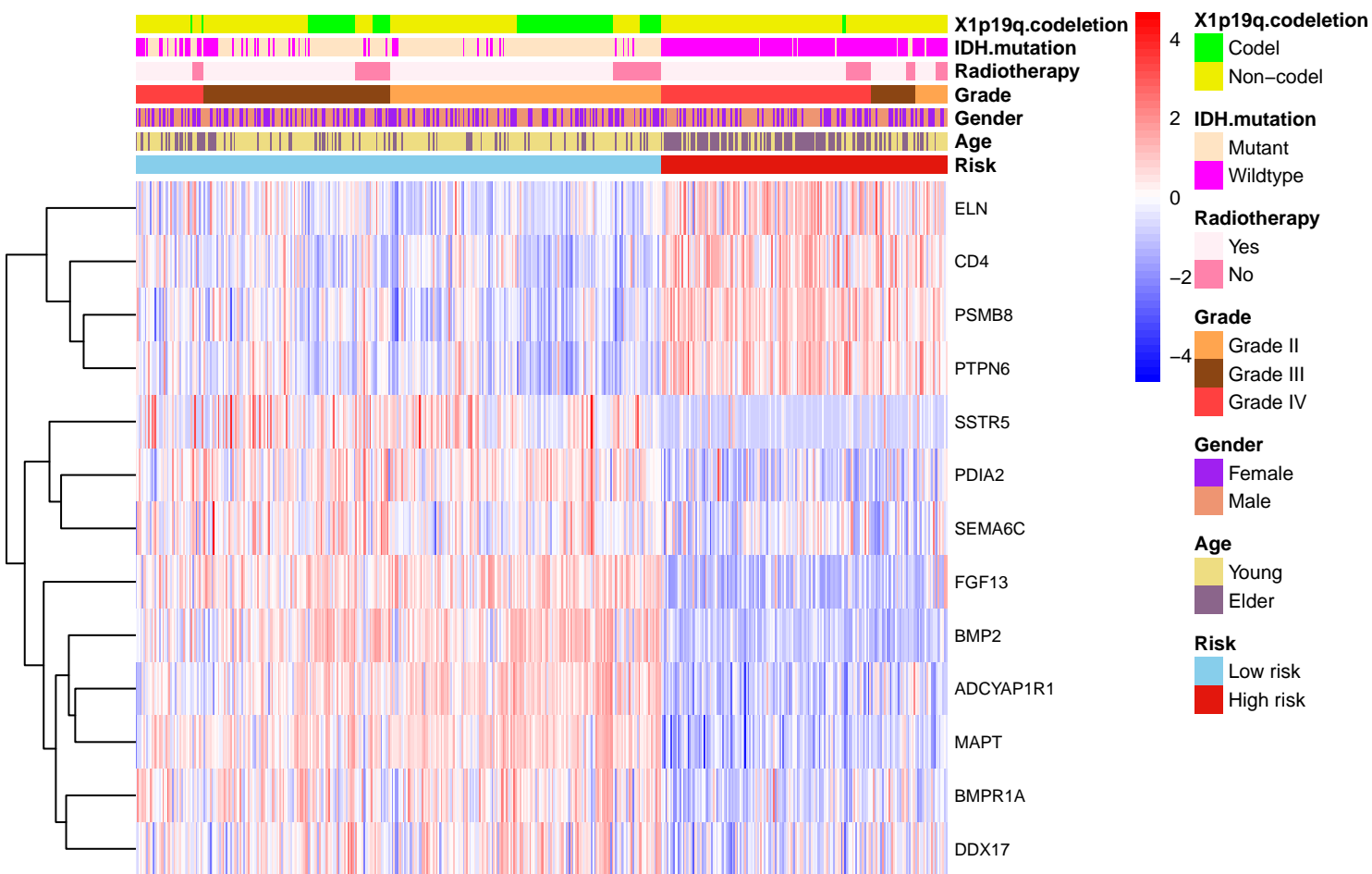

Supplement: Supplementary file 2 [file Image9.PDF]

A

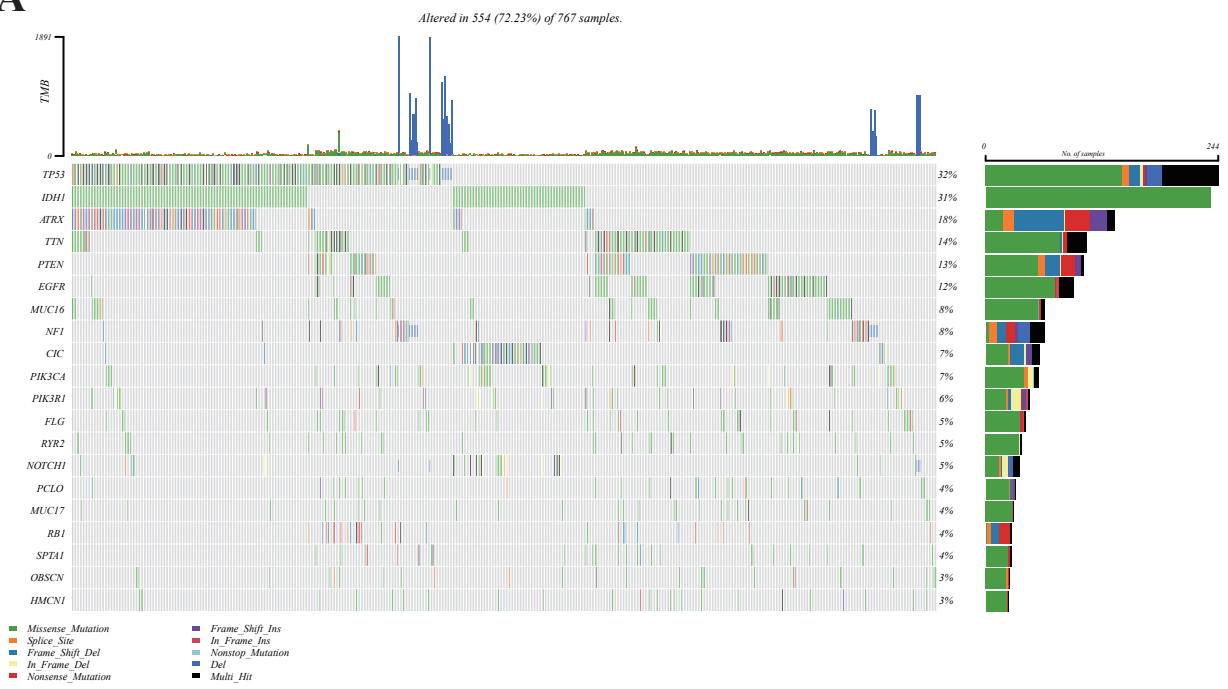

B

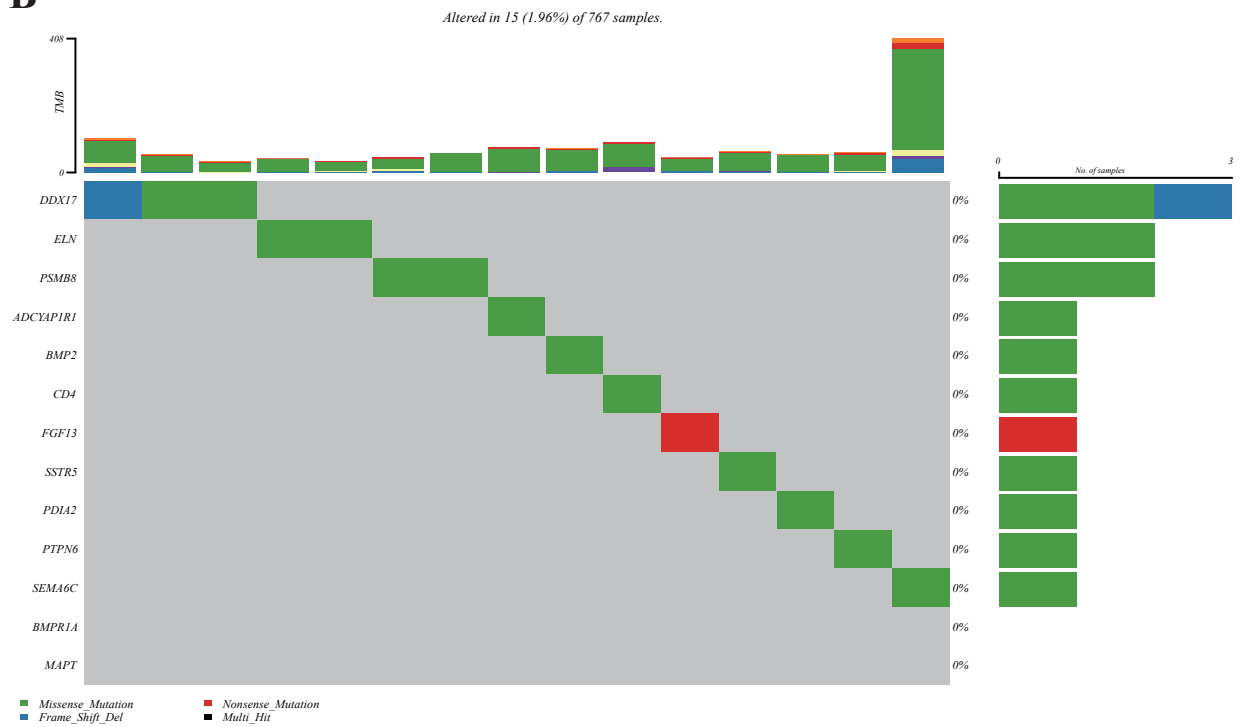

Supplement: Supplementary file 3 [file Image10.PDF]

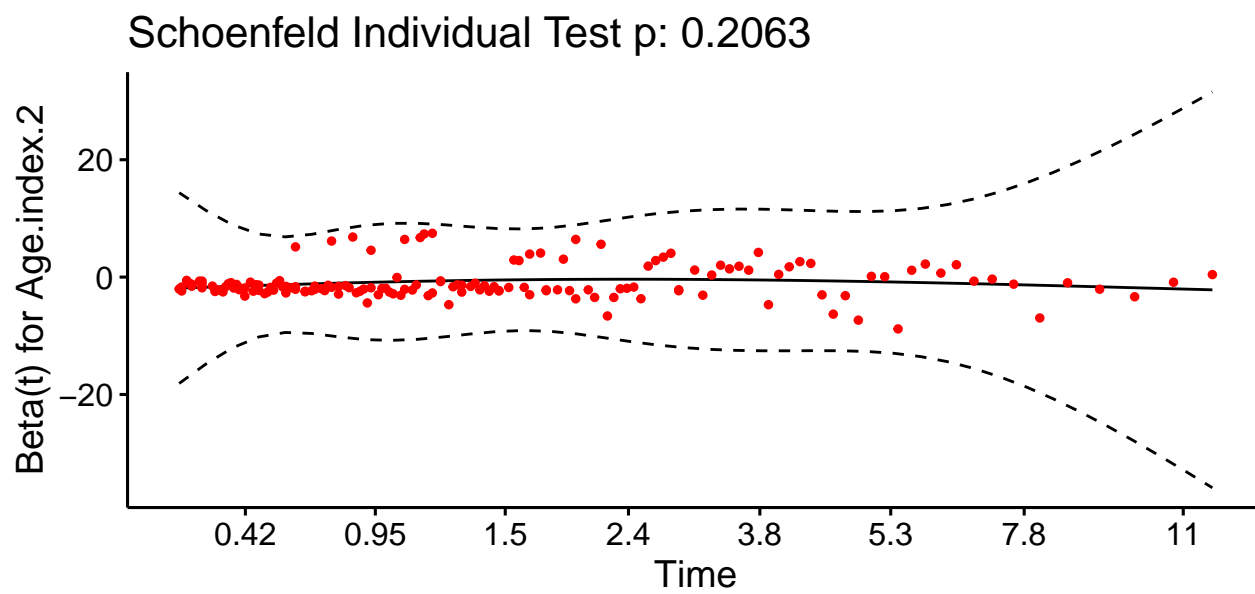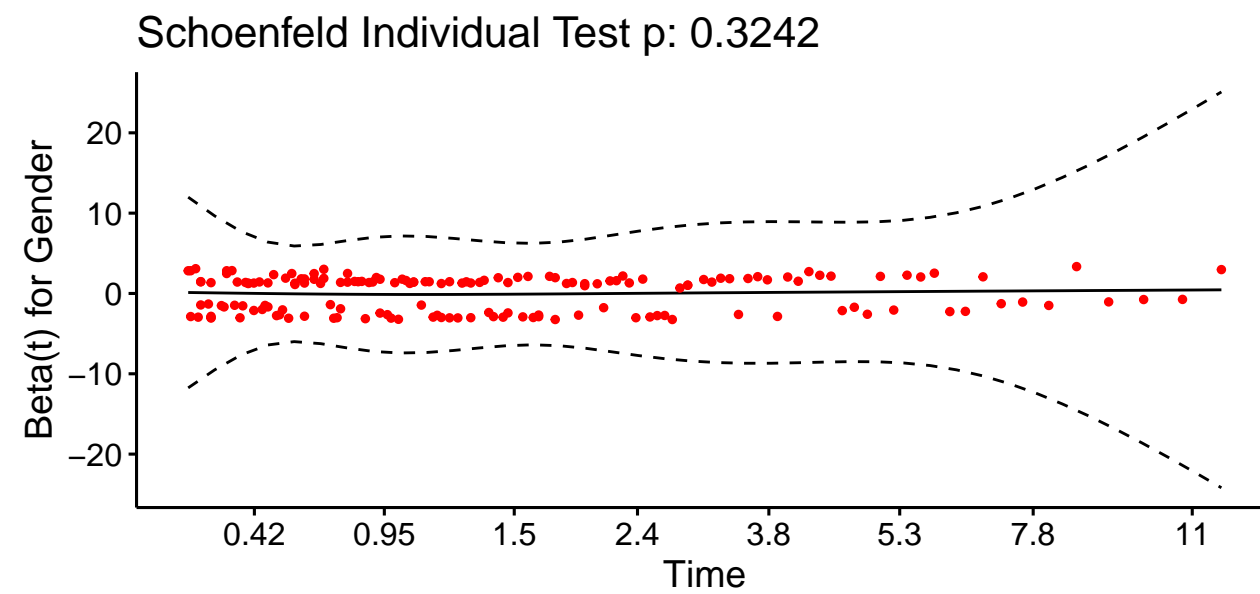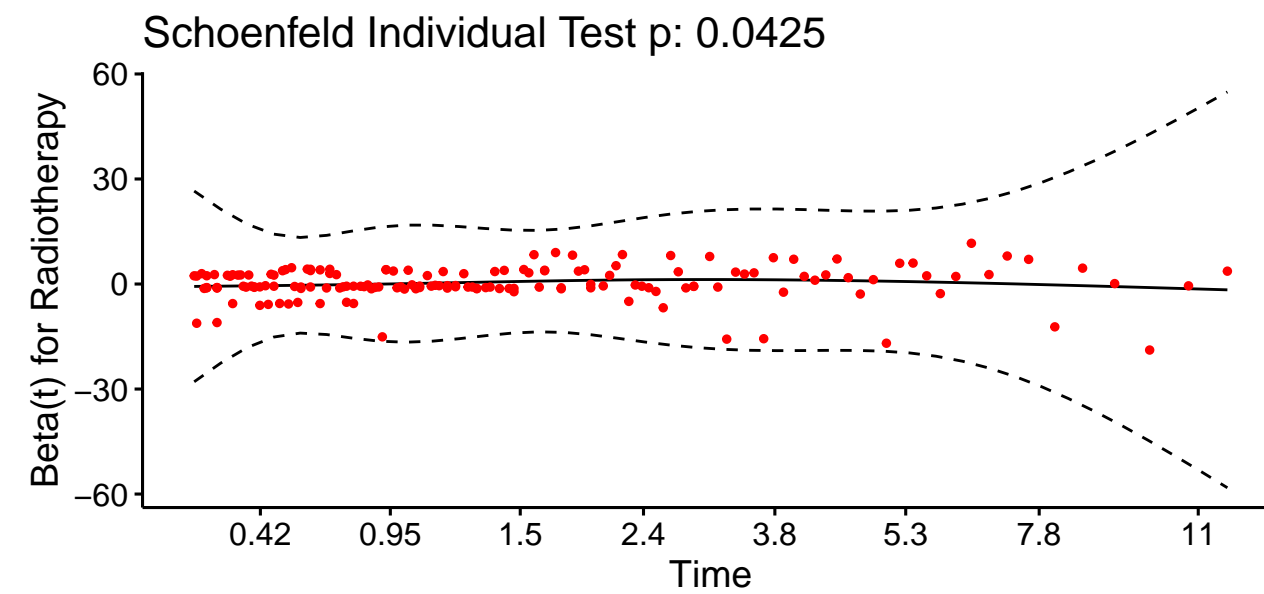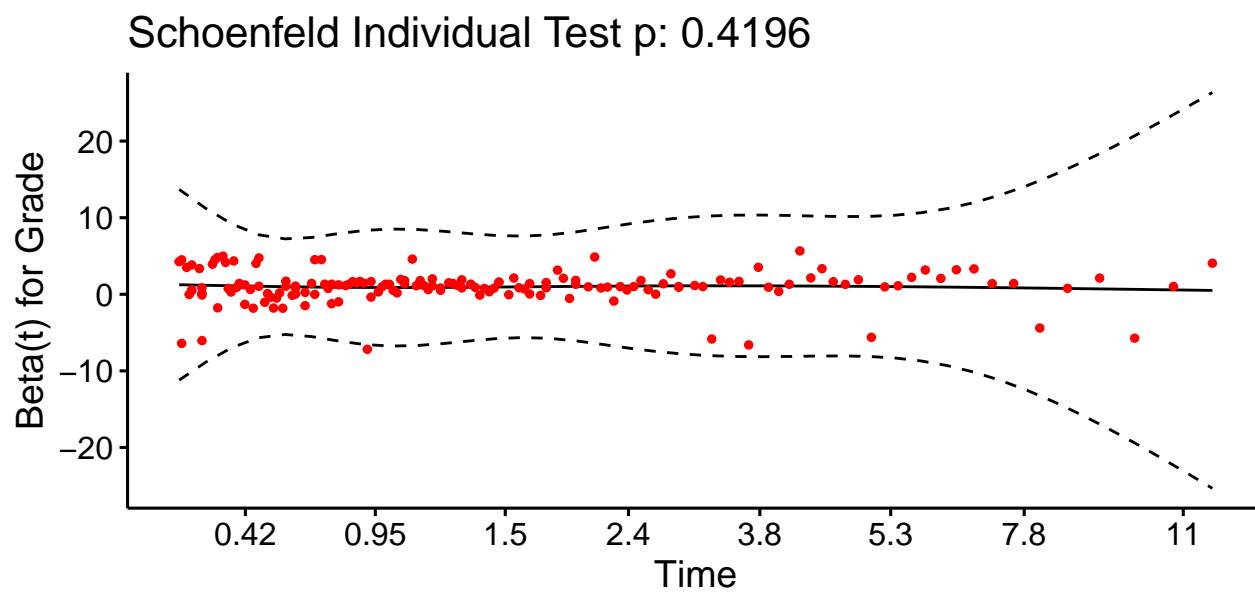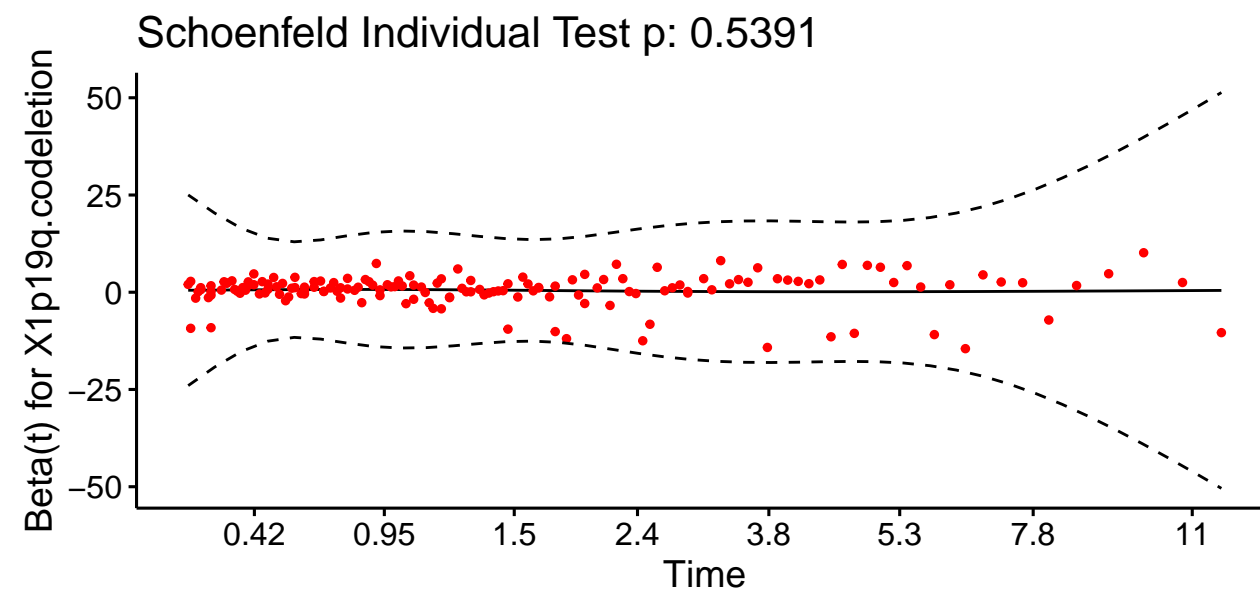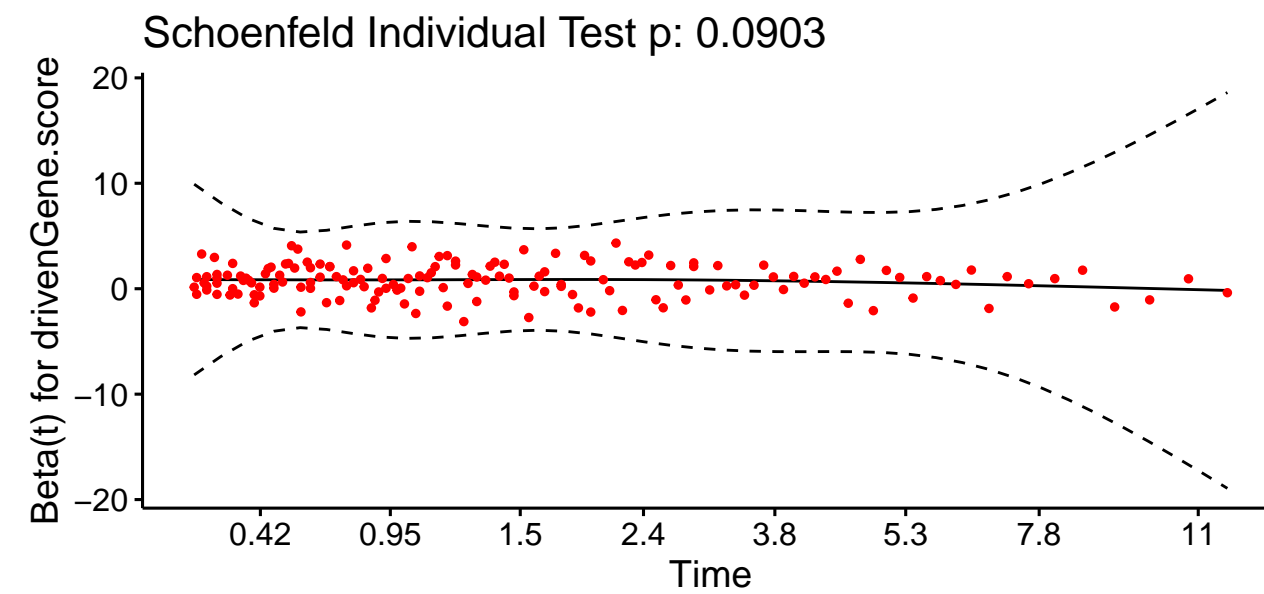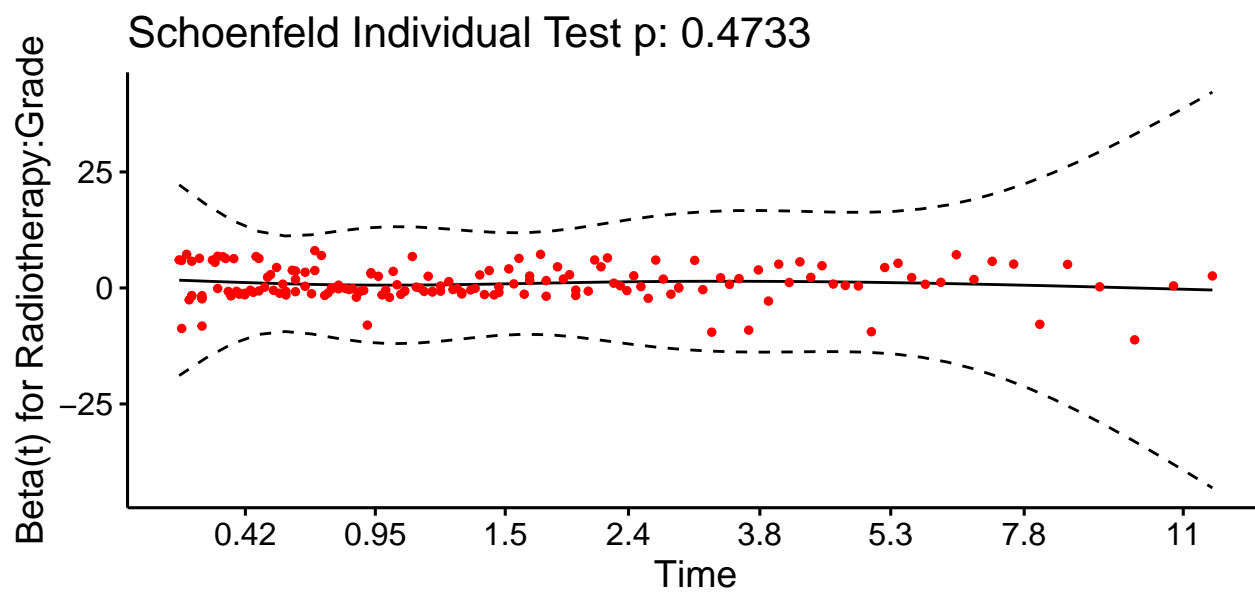

Supplement: Supplementary file 4 [file Image6.PDF]

A

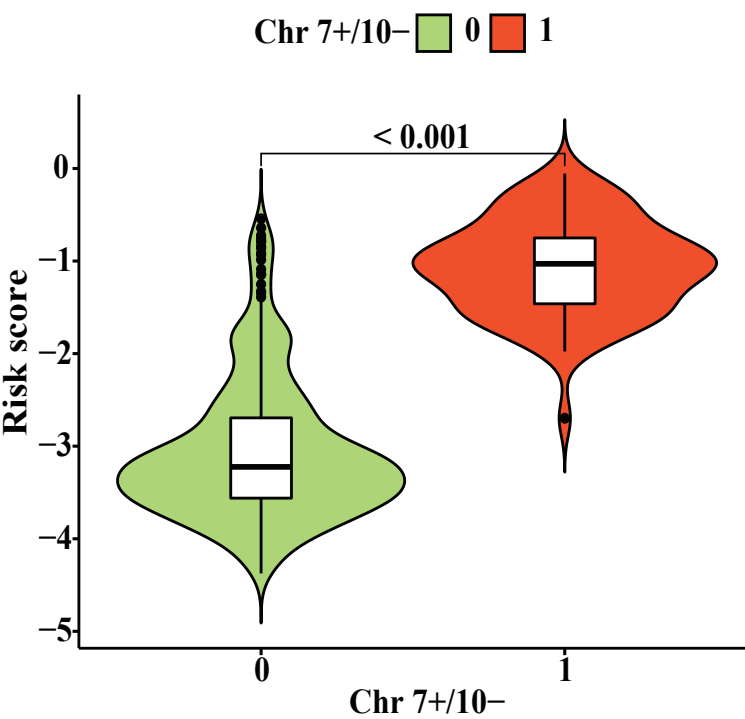

B

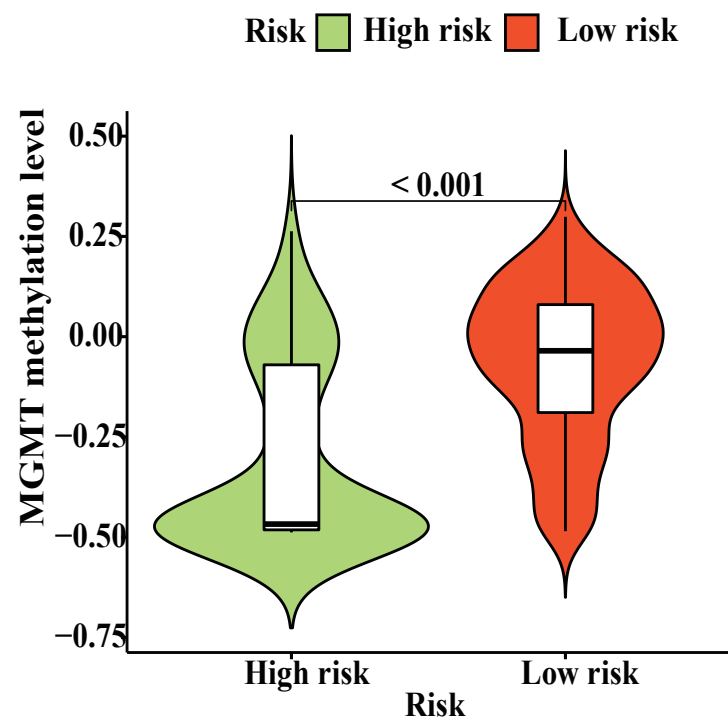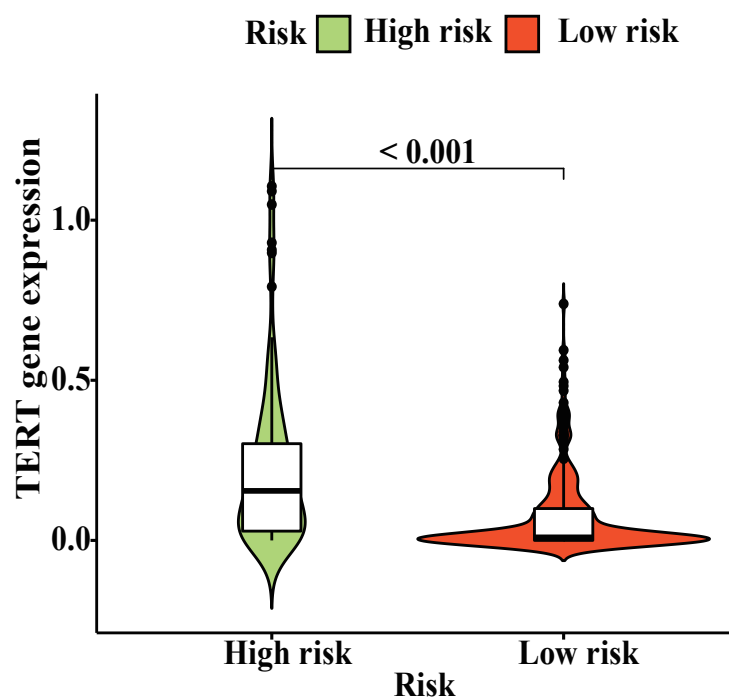

Supplement: Supplementary file 5 [file Image8.PDF]

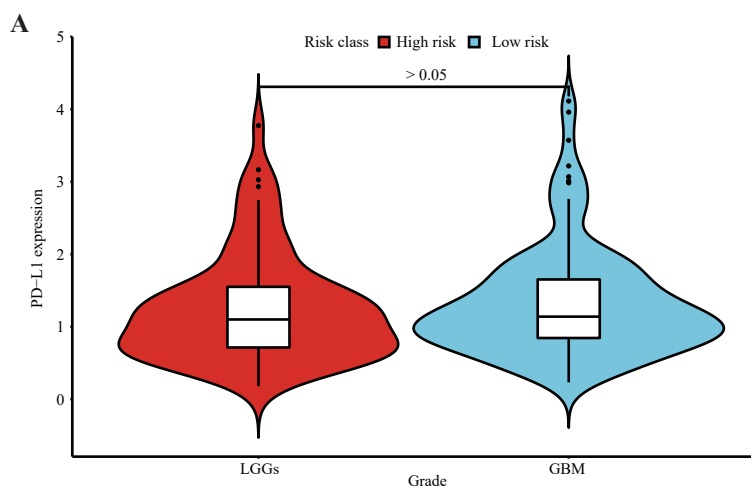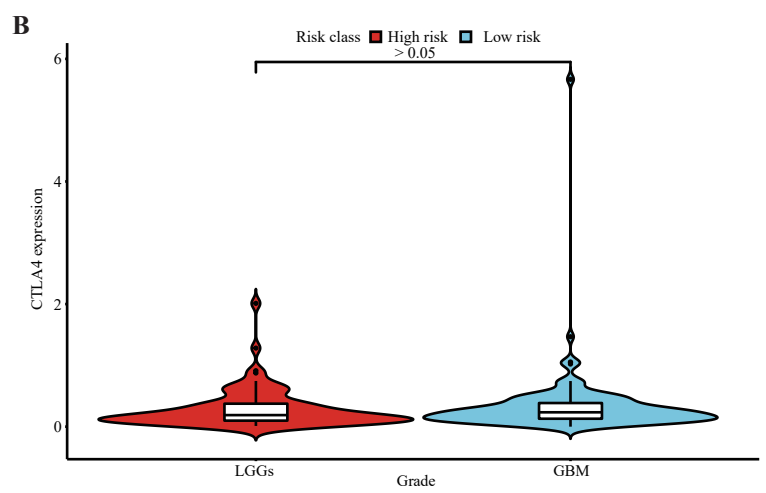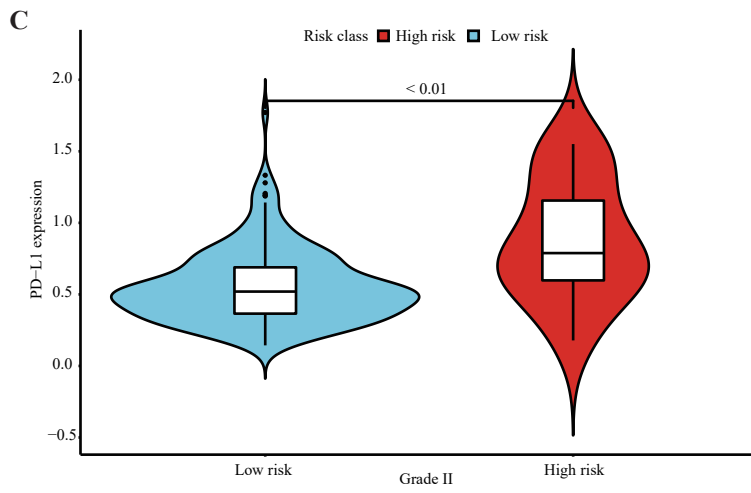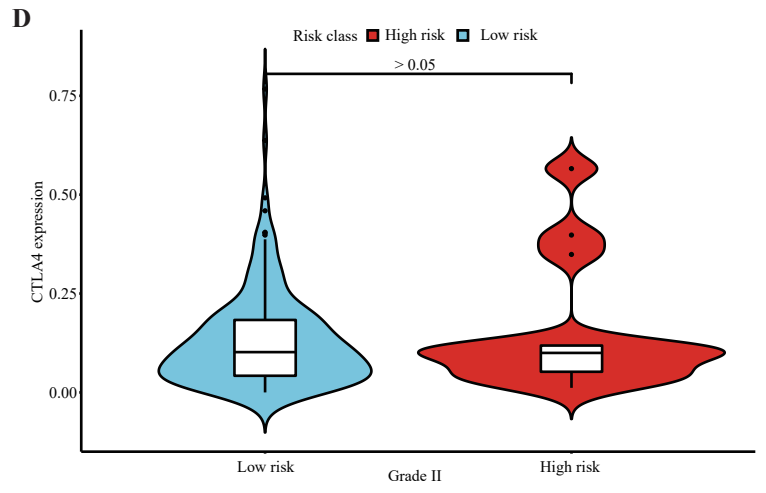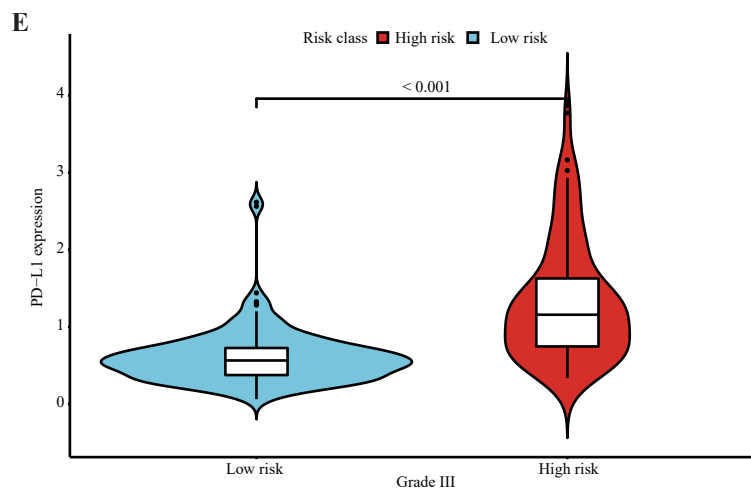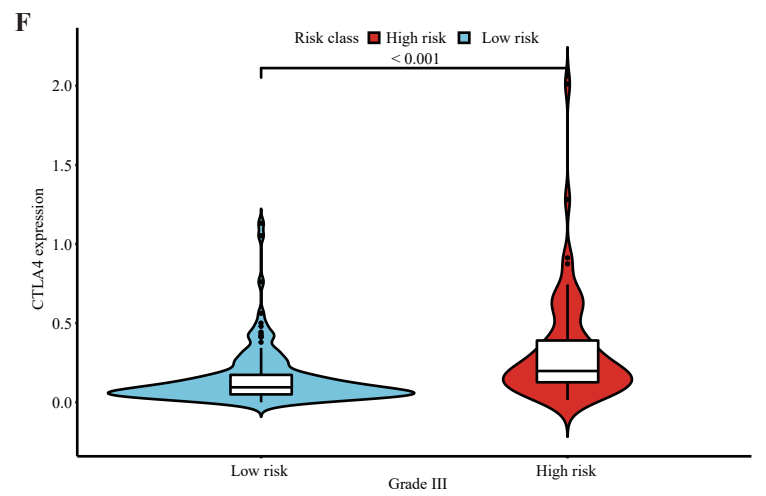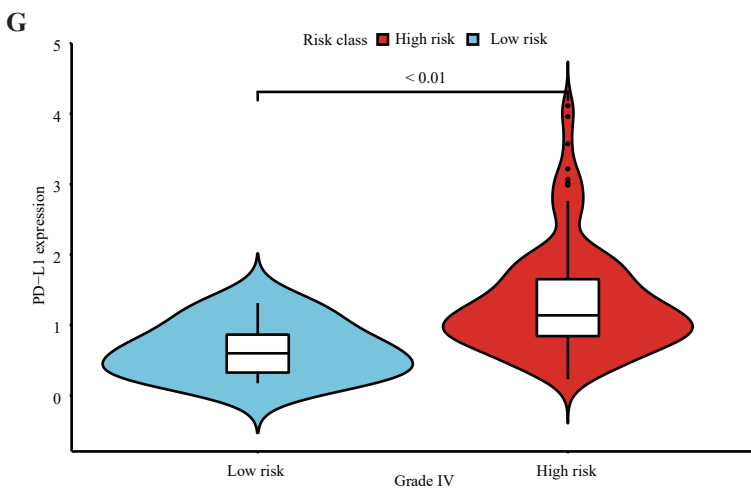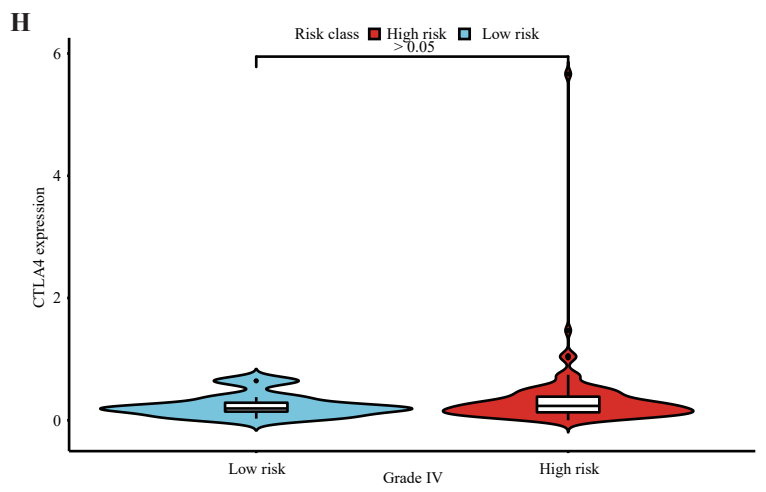

Supplement: Supplementary file 6 [file Image4.PDF]

A

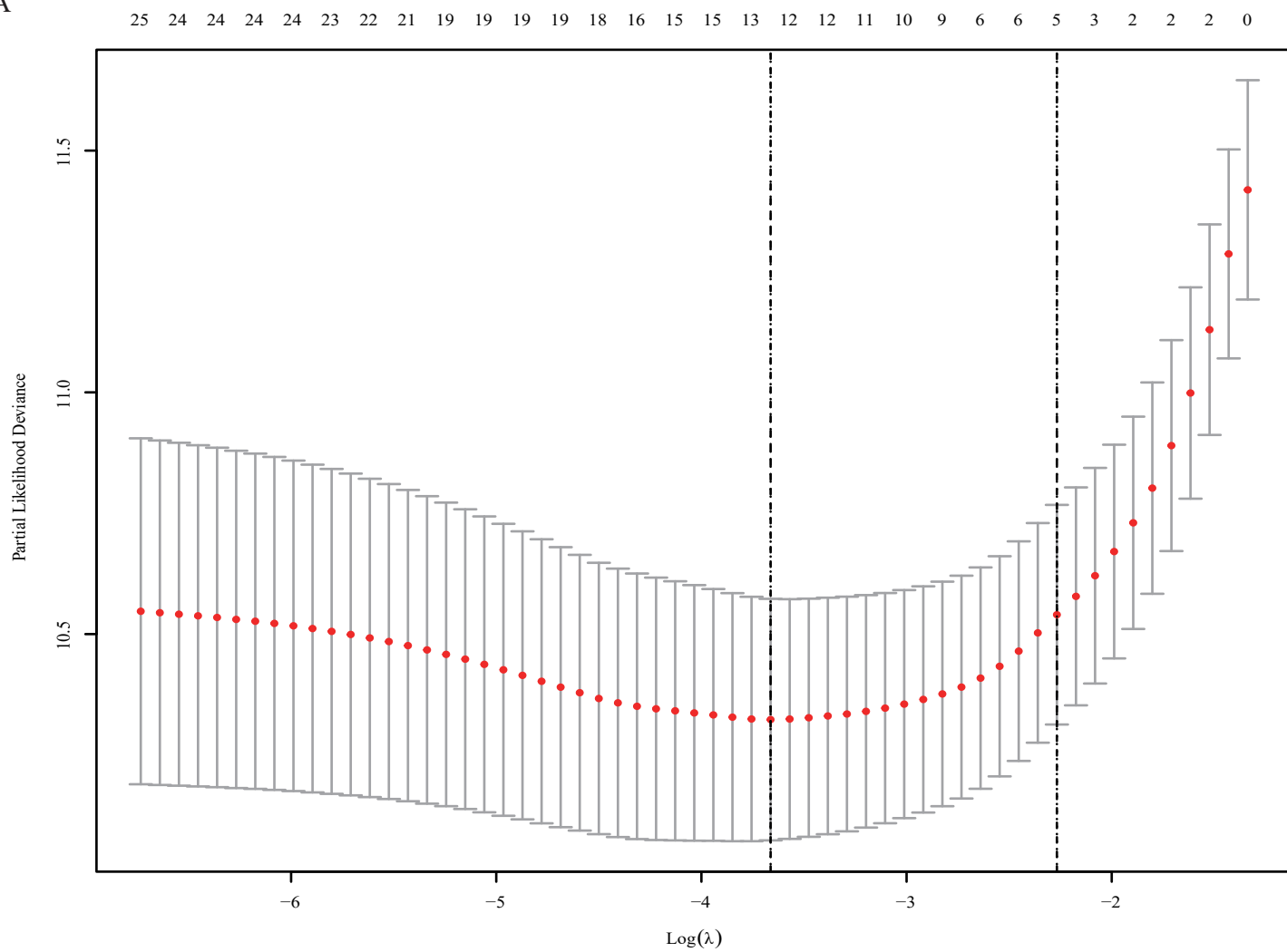

B

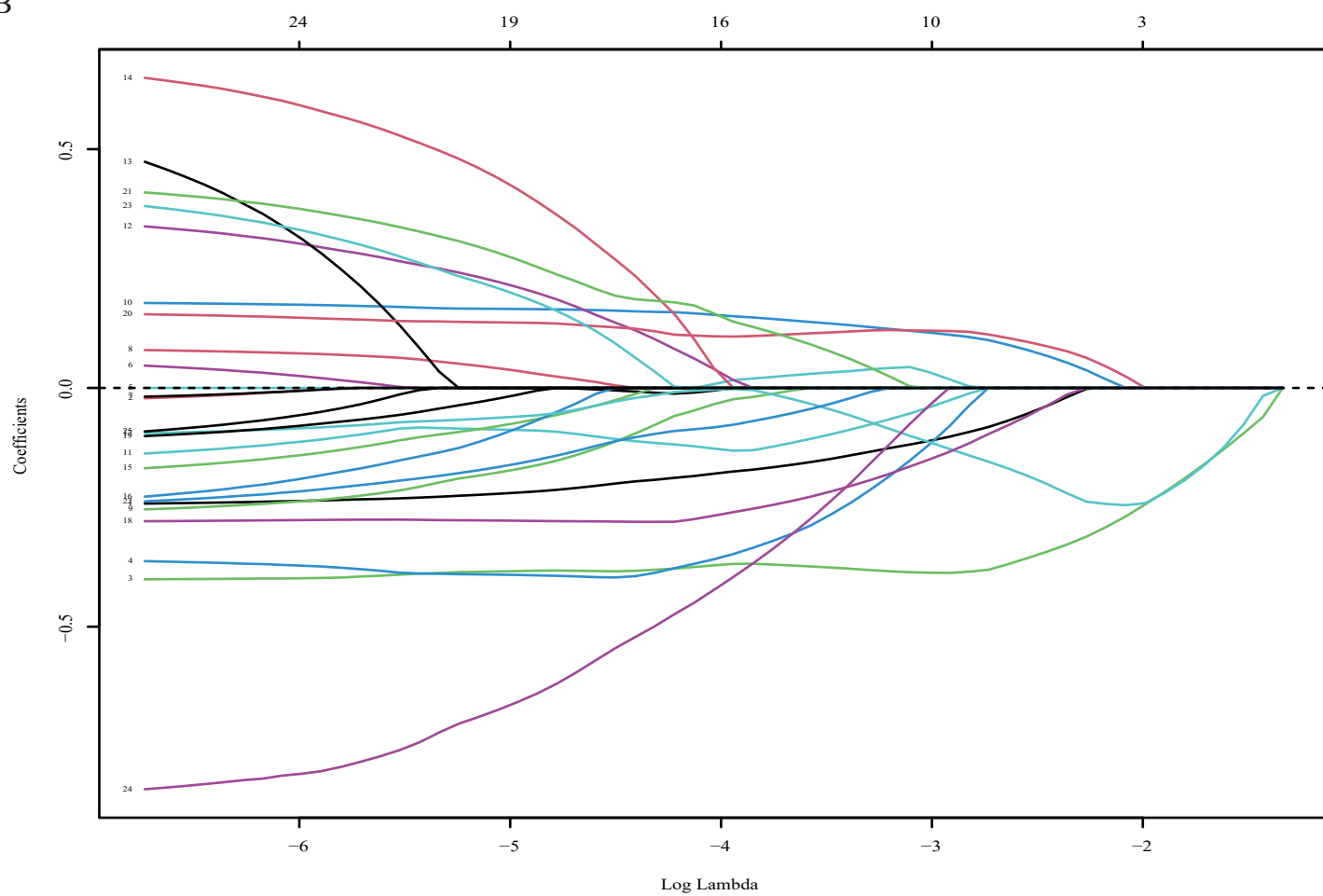

Supplement: Supplementary file 7 [file Image2.PDF]

A

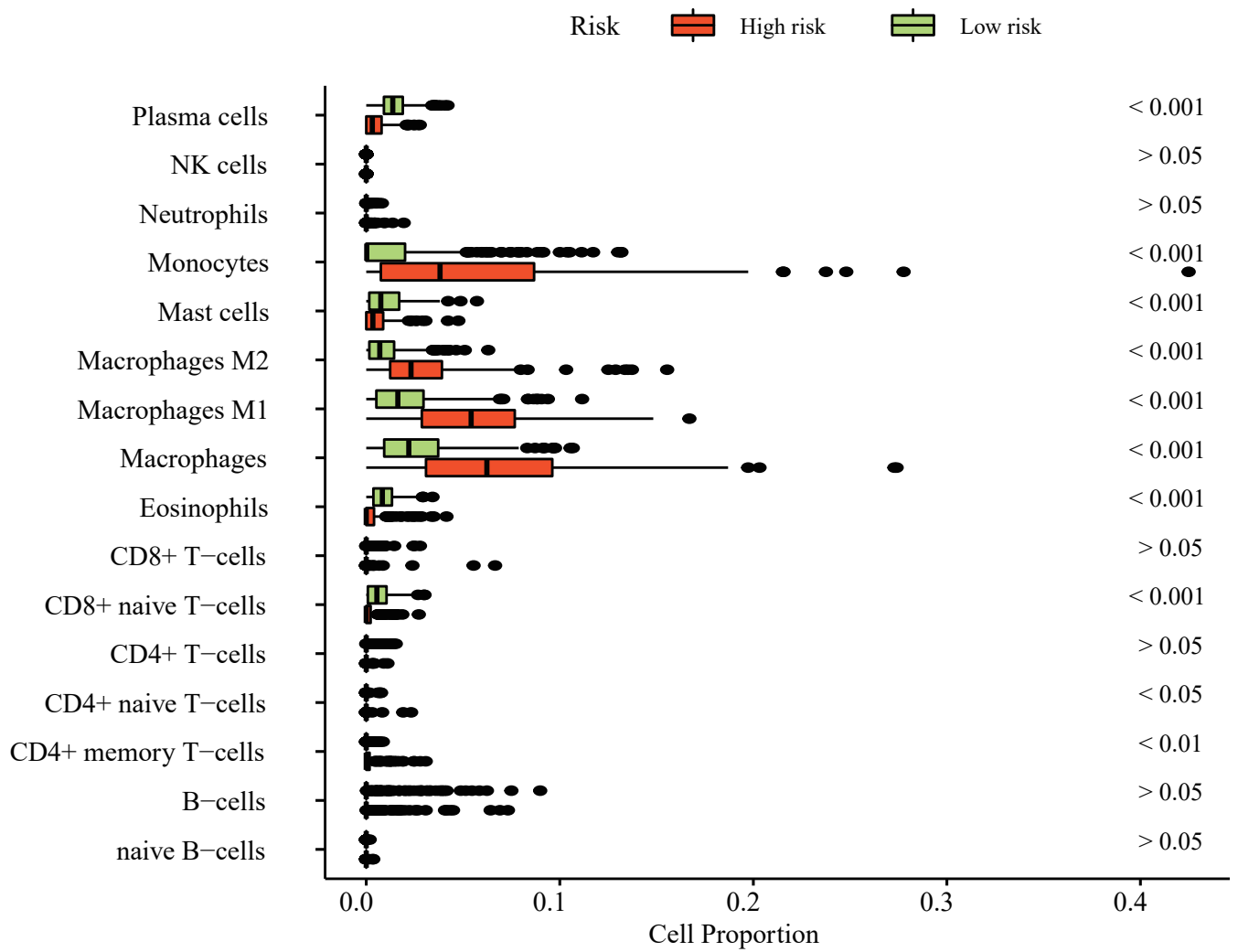

B

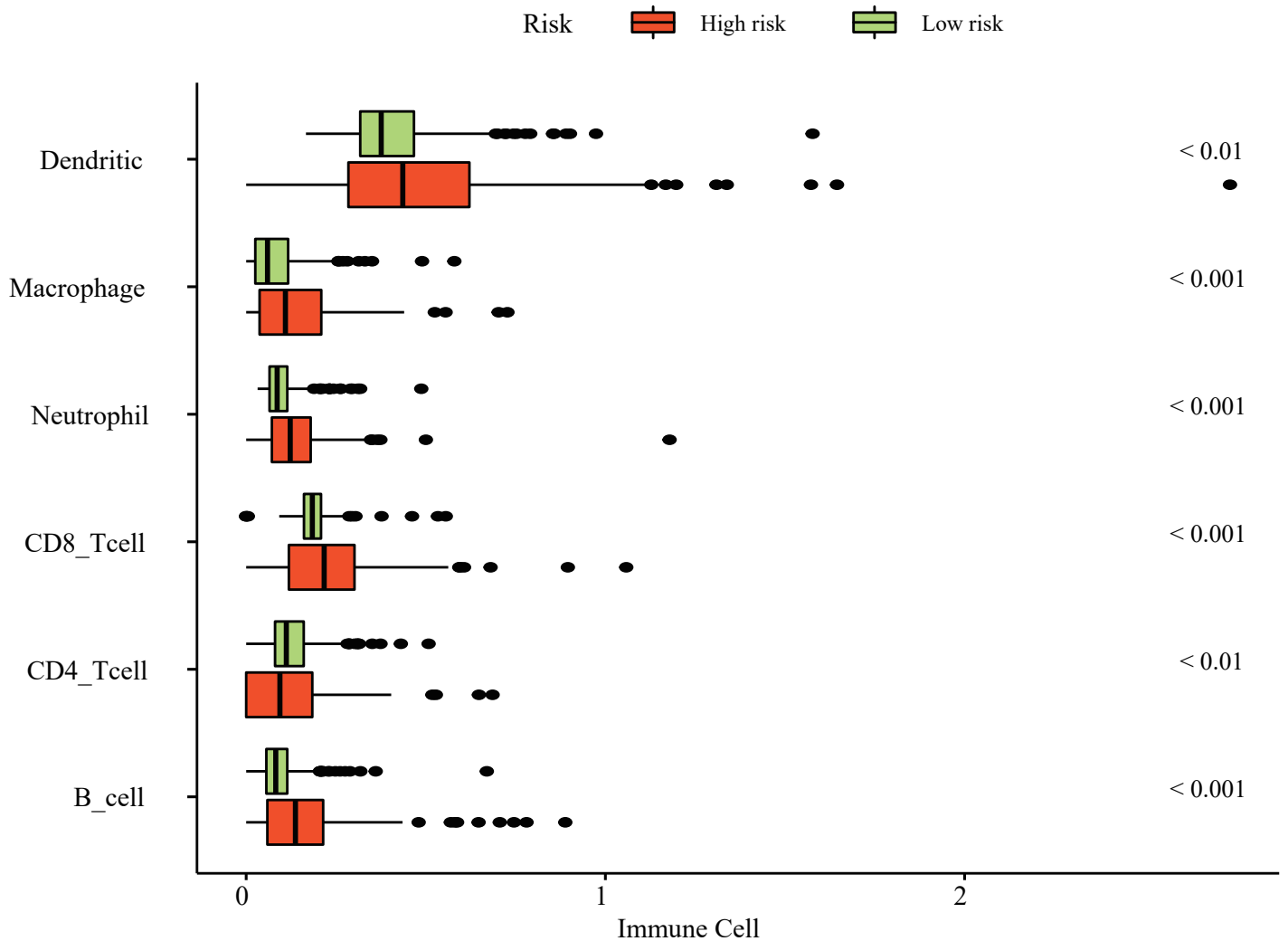

Supplement: Supplementary file 8 [file Image3.PDF]

A

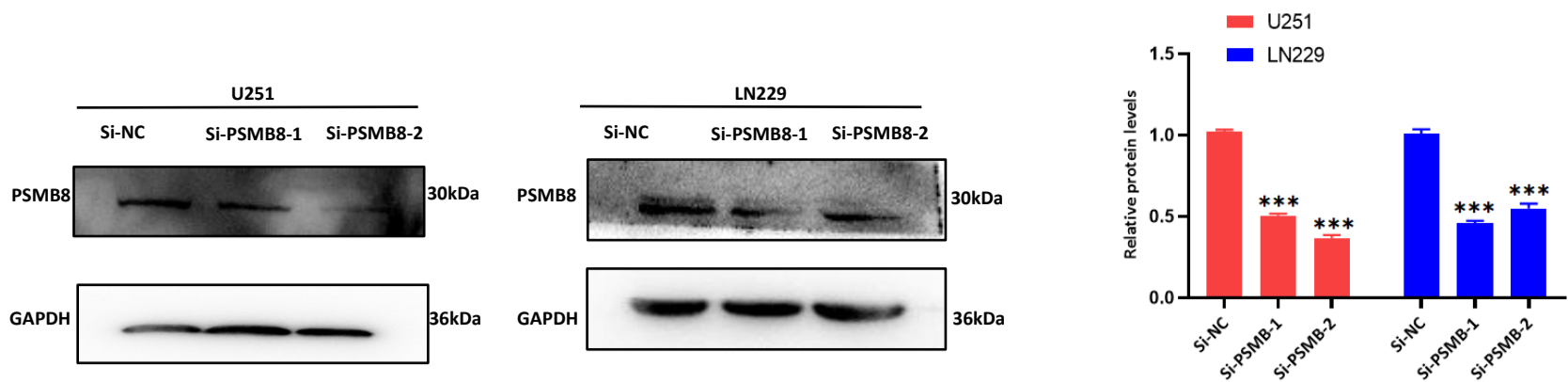

B

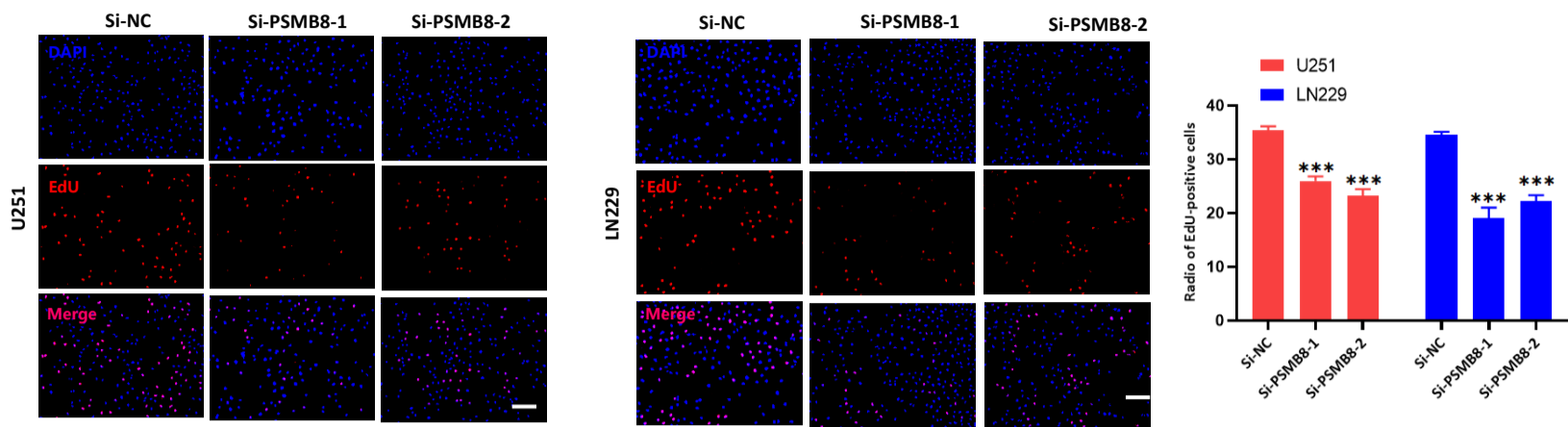

C

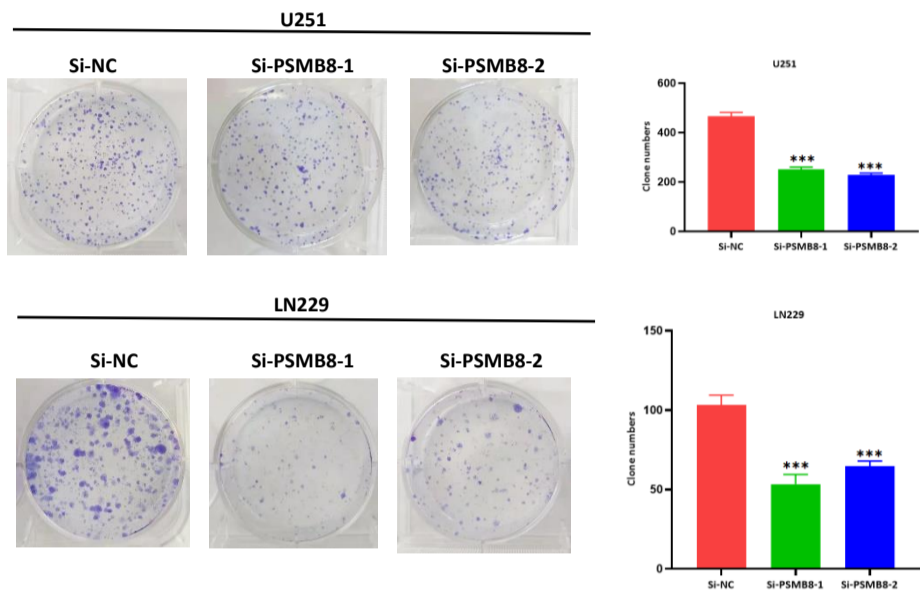

D

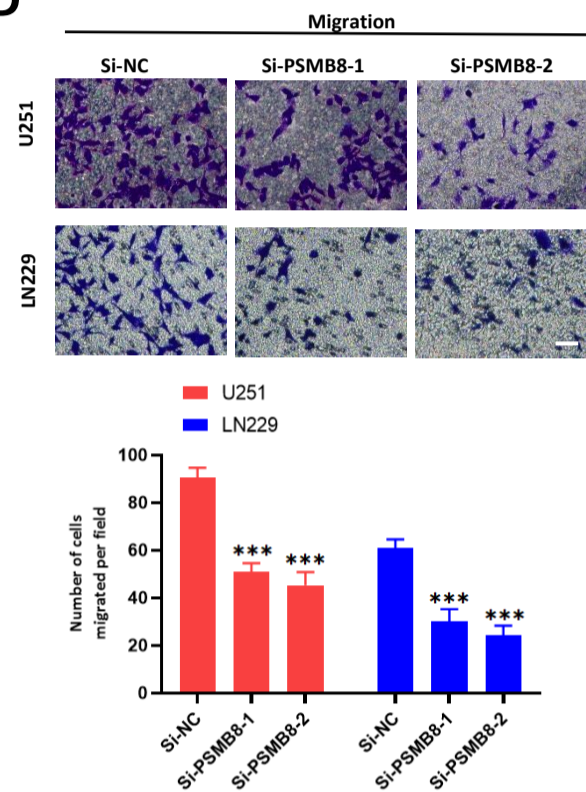

E

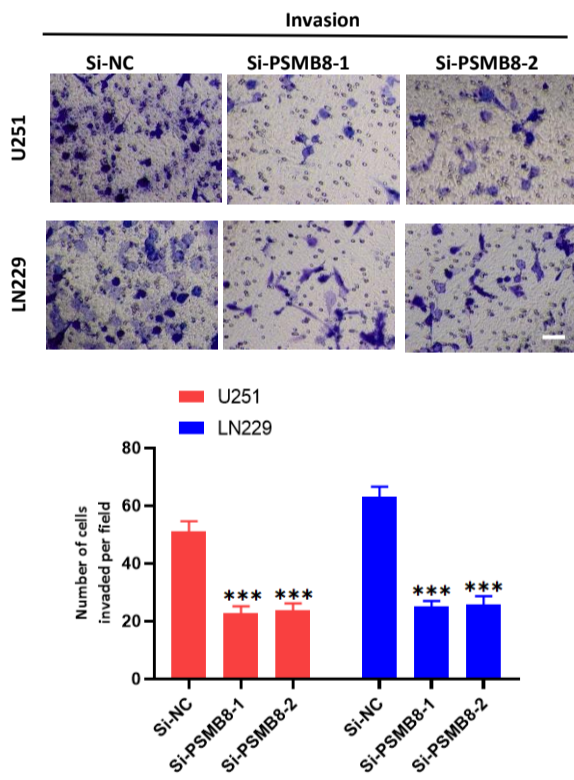

F

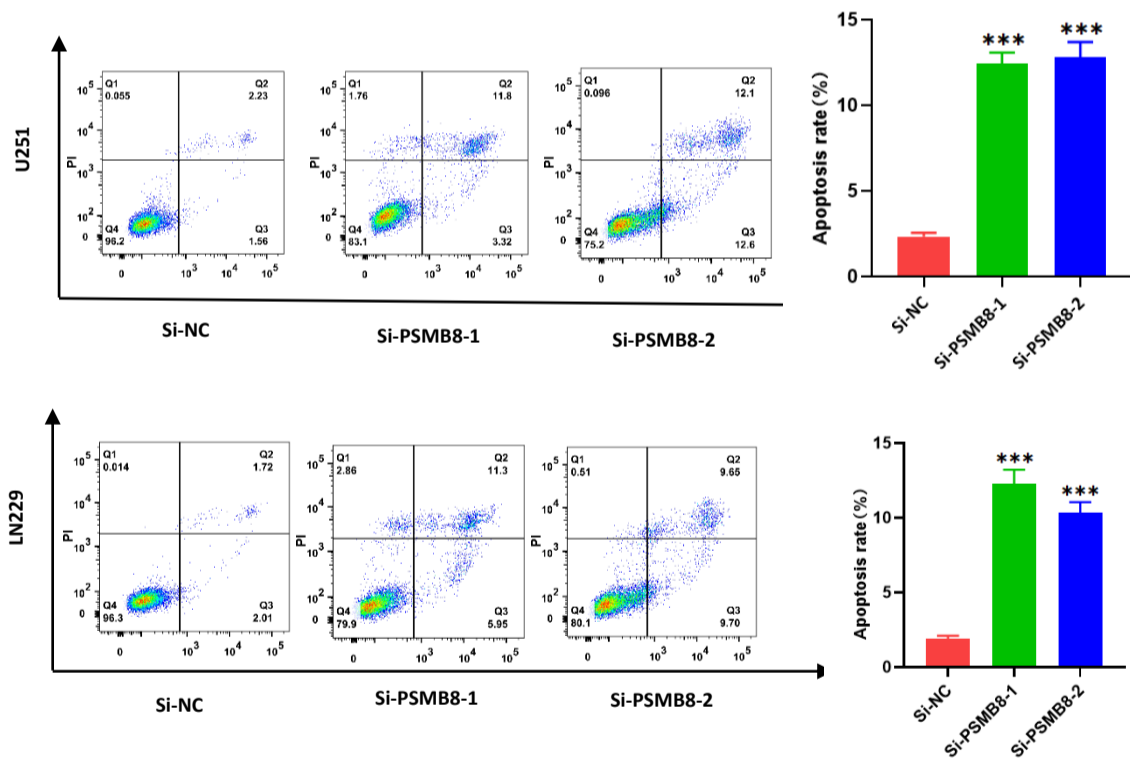

G

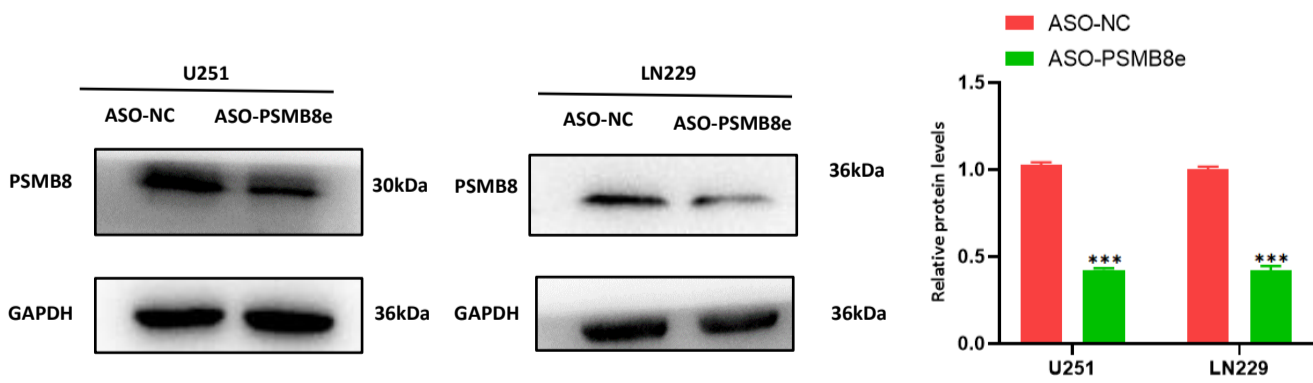

H

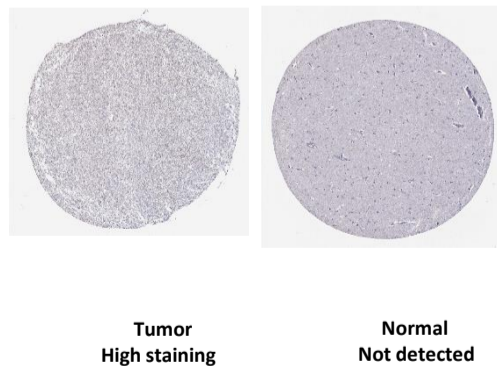

Supplement: Supplementary file 9 [file Image11.PDF]

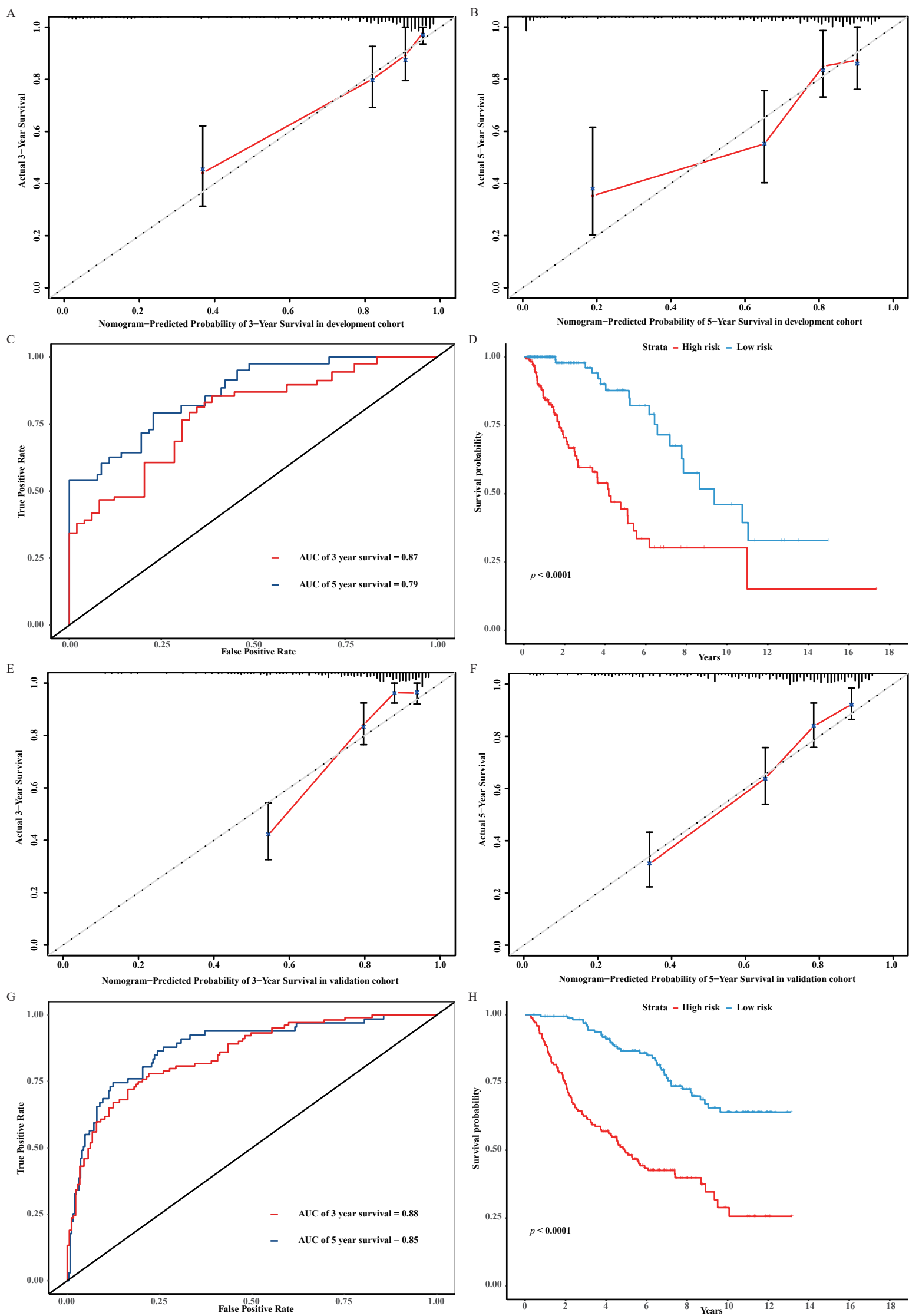

Supplement: Supplementary file 10 [file Image7.PDF]

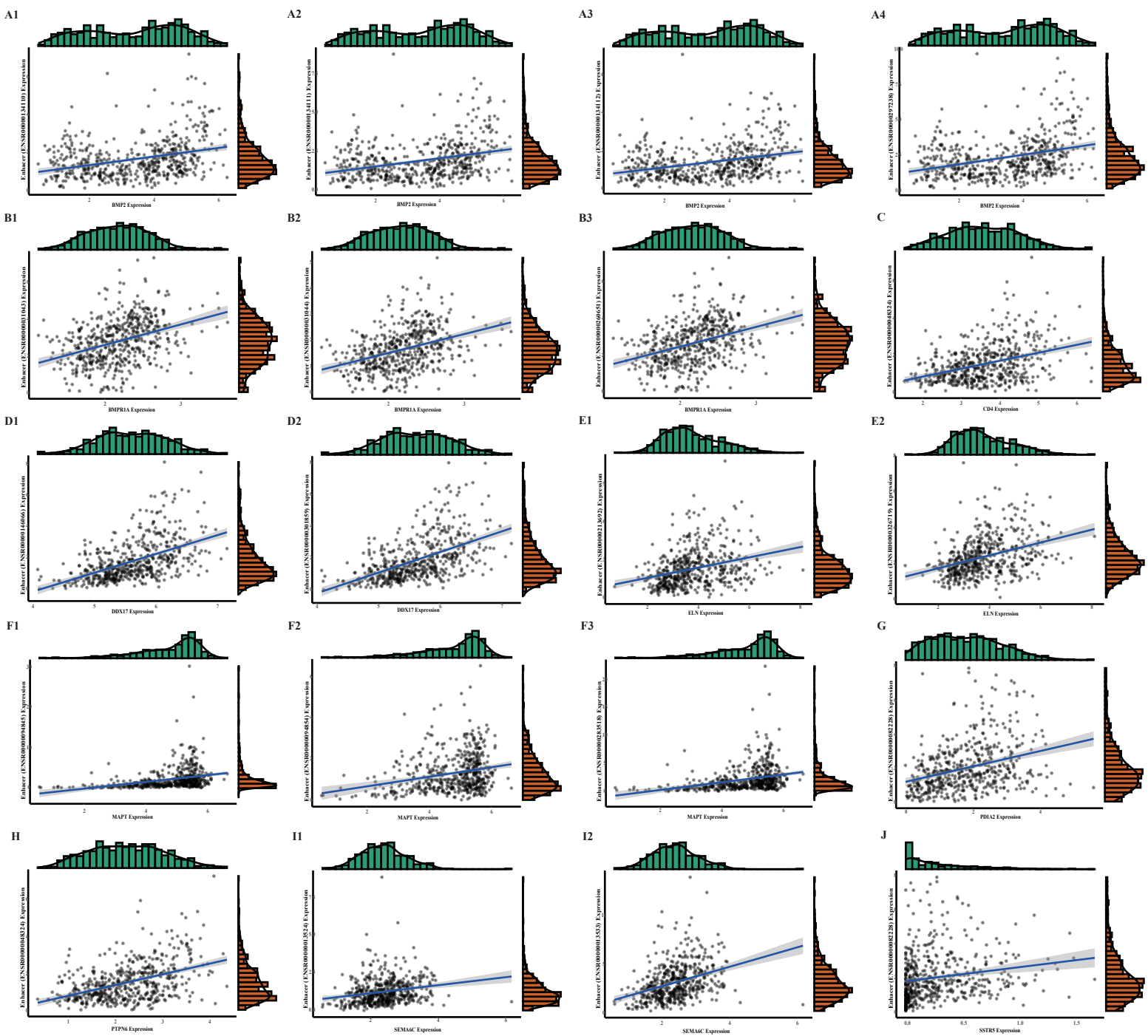

Supplement: Supplementary file 12 [file Image1.PDF]
