# Supplementary material for: A Novel Prognostic Tool for Glioma Based on Enhancer RNA-Regulated Immune Genes
Source: Front Cell Dev Biol. 2022 Jan 20;9:798445. doi: 10.3389/fcell.2021.798445 (PMC8811171; doi:10.3389/fcell.2021.798445)
Supplement: Supplementary file 11 [file DataSheet1.DOCX]

We have uploaded the data to <https://www.jianguoyun.com/p/Db9O3rAQ0cDrBxi37JUE>. You can freely downloaded the original data.
